# Disentangling Heterogeneous Molecular Networks for Multi‐Omics‐Driven Cancer Driver Discovery

**DOI:** 10.1002/advs.77970

**Published:** 2026-09-27

**Authors:** Xinjing Gong, Ji Li, Mu Su, Peishen Yu, Te Ma, Ruiyang Zhai, Chenye Zhang, Mengyan Zhang, Yan Zhang

**Affiliations:** ^1^ School of Life Science and Technology, Faculty of Life Sciences and Medicine Harbin Institute of Technology Harbin China; ^2^ School of Intelligent Medicine and Technology (Big Data Research Center) Hainan Medical University Haikou China

**Keywords:** cancer driver genes, contrastive learning, graph representation learning, multi‐omics integration, precision oncology, protein–protein interaction networks

## Abstract

Prioritizing cancer driver genes amid passenger alterations remains challenging because protein–protein interaction (PPI) networks are heterophilic, multi‐omics evidence is heterogeneous and can conflict, and driver annotations are sparse. DRIVE is a semi‐supervised graph framework integrating mutation frequency, copy‐number aberration, DNA methylation, and gene expression with biological networks. It separates PPI neighborhoods into tight and loose semantic views based on learnable representation consistency, reducing cross‐class signal mixing. Multi‐omics evidence is decomposed into omics‐common and omics‐specific components through contrastive mutual‐information learning and the soft orthogonality constraint. Joint training combines self‐supervised learning with focal and max‐margin objectives to improve prioritization under sparse, imbalanced annotations. Across six benchmark PPI networks, DRIVE outperforms ten methods, achieving mean areas under the precision–recall curve (AUPRC) and receiver operating characteristic curve (AUROC) of 0.9204 and 0.9704, respectively. Ablation, representation, and masked‐driver recovery analyses show that DRIVE captures complementary network and molecular signals and remains robust to incomplete annotations. DRIVE identifies 186 high‐confidence candidate driver genes enriched near known drivers, 80.1% of which receive DepMap CRISPR dependency support. These candidates reveal underappreciated connections to tumor regulatory programs, particularly NF‐κB‐associated inflammation, T‐cell activation, and immune checkpoint regulation. Pharmacogenomic associations further suggest therapeutic vulnerabilities.

## Graphical abstract

DRIVE integrates PPI topology and pan‐cancer multi‐omics profiles through dual‐view graph disentanglement, contrastive representation learning, and joint optimization. Across six PPI networks, DRIVE outperforms ten baselines and remains robust to structural and annotation perturbations. The resulting 186 candidate driver genes are supported by genomic evidence, CRISPR dependency, immune regulation, and therapeutic response associations.

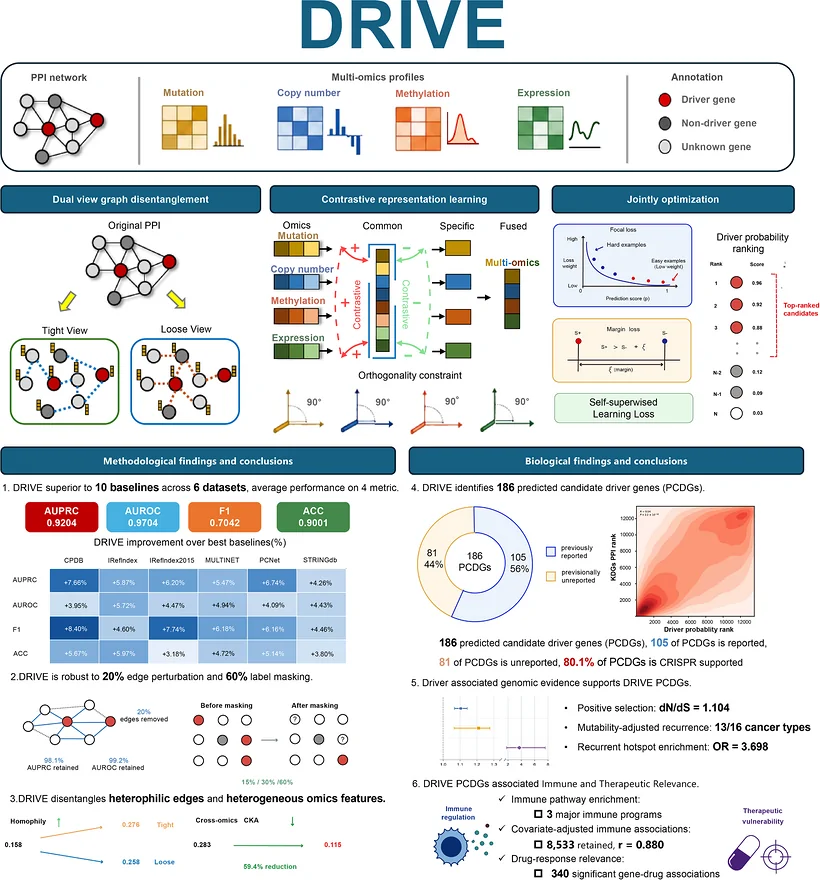

## 1 Introduction

Cancer driver genes carry molecular alterations that confer selective growth advantages and promote malignant phenotypes [1, 2, 3, 4, 5, 6]. Their accurate identification is therefore essential for understanding tumor evolution, interpreting patient genomes, and prioritizing targets for precision oncology. Large‐scale cancer genomics programs, including the International Cancer Genome Consortium and The Cancer Genome Atlas, have generated extensive molecular profiles across tumor types [7, 8]. Curated resources such as the Network of Cancer Genes and the COSMIC Cancer Gene Census have cataloged many drivers [9, 10], but these catalogs remain incomplete. Many drivers occur at low mutation frequencies outside specific cancer types [11], and driver activity can also arise through non‐mutational events, including copy‐number alteration and aberrant DNA methylation [12, 13]. Because driver genes also act through pathway and protein‐interaction contexts [14], effective prioritization requires models that integrate molecular abnormalities with biological networks rather than relying on any single data source.

Computational driver‐gene discovery has moved from feature engineering to graph‐based learning [14, 15]. Earlier machine‐learning methods encoded mutation, copy‐number aberration, DNA methylation and gene expression as engineered features, sometimes augmented with network embeddings, and then applied classifiers or ranking models such as support vector machines, random forests and XGBoost [16, 17, 18, 19, 20]. A second line of work mapped molecular data onto biological networks and used propagation or random‐walk methods, including DeepWalk, node2vec, PageRank and diffusion, to infer genes in functional modules or pathways [21, 22, 23, 24]. These approaches established the value of combining omics and networks, but they had limited capacity to learn nonlinear interactions between molecular evidence and graph topology in an end‐to‐end manner. Graph neural networks provide a more direct representation of this problem by treating genes as nodes, PPIs as edges, and multi‐omics profiles as node attributes [25, 26]. EMOGI coupled pan‐cancer multi‐omics features with PPI networks through graph convolution and used the resulting node embeddings for driver‐gene classification [27]. Subsequent models improved this paradigm by increasing graph expressivity, adding multi‐omics fusion strategies or introducing auxiliary training objectives. For example, GRAFT and TREE used Transformer architectures to capture long‐range dependencies [28, 29], SGCD introduced feature separation and residual connections in heterophilic networks [30], deepCDG encoded omics‐specific features before attention‐based fusion [31], and MODIG and MODCAN incorporated additional gene‐network relationships [32, 33]. MTGCN and SMG further showed that auxiliary graph objectives can improve representation learning under limited supervision [34, 35].

Despite these advances, three linked obstacles still limit graph representation learning for driver‐gene prioritization. First, PPI networks are strongly heterophilic: interacting proteins often have different driver status, so direct graph propagation can mix signals from driver and non‐driver genes. Second, multi‐omics features are heterogeneous and context‐dependent. Mutation, copy‐number aberration, DNA methylation, and gene expression can provide complementary or conflicting evidence, and simple concatenation can compress redundant or discordant signals into a less discriminative representation. Third, supervision is sparse and imbalanced because known drivers represent only a small fraction of protein‐coding genes, reliable non‐driver labels are limited, and many genes remain unlabeled. These conditions can bias supervised models toward well‐studied drivers and reduce their ability to prioritize previously unrecognized candidates.

To address these challenges, we developed DRIVE (Disentangled gRaph representation learnIng with contrastiVe multi‐omics fusion for cancEr driver gene identification), a semi‐supervised graph learning framework for cancer driver prioritization. DRIVE constructs omics‐level gene graphs by integrating multi‐omics evidence with PPI networks. First, each gene graph is dynamically partitioned into tight and loose views according to learnable representation consistency, and the two views are subsequently aggregated through view‐oriented attention to obtain omics‐level gene representations. These representations are then further decomposed into omics‐common and omics‐specific components, with contrastive learning and a soft orthogonality constraint reducing cross‐omics redundancy. Finally, a multi‐objective optimization strategy combining supervised and self‐supervised losses is used to train the model under sparse and imbalanced driver annotations, and unlabeled genes are prioritized according to their predicted driver probabilities. We systematically evaluated DRIVE across diverse PPI networks and benchmarked it against representative computational approaches. Extensive computational analyses and independent biological validations demonstrated the robustness and generalizability of DRIVE for prioritizing candidate cancer driver genes. Furthermore, DRIVE identified high‐confidence candidate drivers supported by network proximity to known cancer genes, functional dependency evidence and associations with tumor regulatory programs and therapeutic vulnerabilities.

## 2 Results

### 2.1 Overview of DRIVE

Graph‐based driver‐gene prediction can integrate molecular alterations with PPI networks, but three properties of the task complicate representation learning. PPI networks are heterophilic because interacting proteins need not share driver status; direct neighborhood aggregation can therefore mix driver and non‐driver signals. Multi‐omics evidence is also gene‐dependent: mutation frequency (MF), copy‐number aberration (CNA), DNA methylation (METH), and gene expression (GE) capture different molecular processes whose contributions vary across genes and cancer contexts. Finally, driver annotations are sparse and imbalanced, with known driver genes (KDGs) representing only a small subset of protein‐coding genes and many genes remaining unlabeled. DRIVE was designed around these three constraints rather than treating them as downstream training artifacts.

**FIGURE 1:**
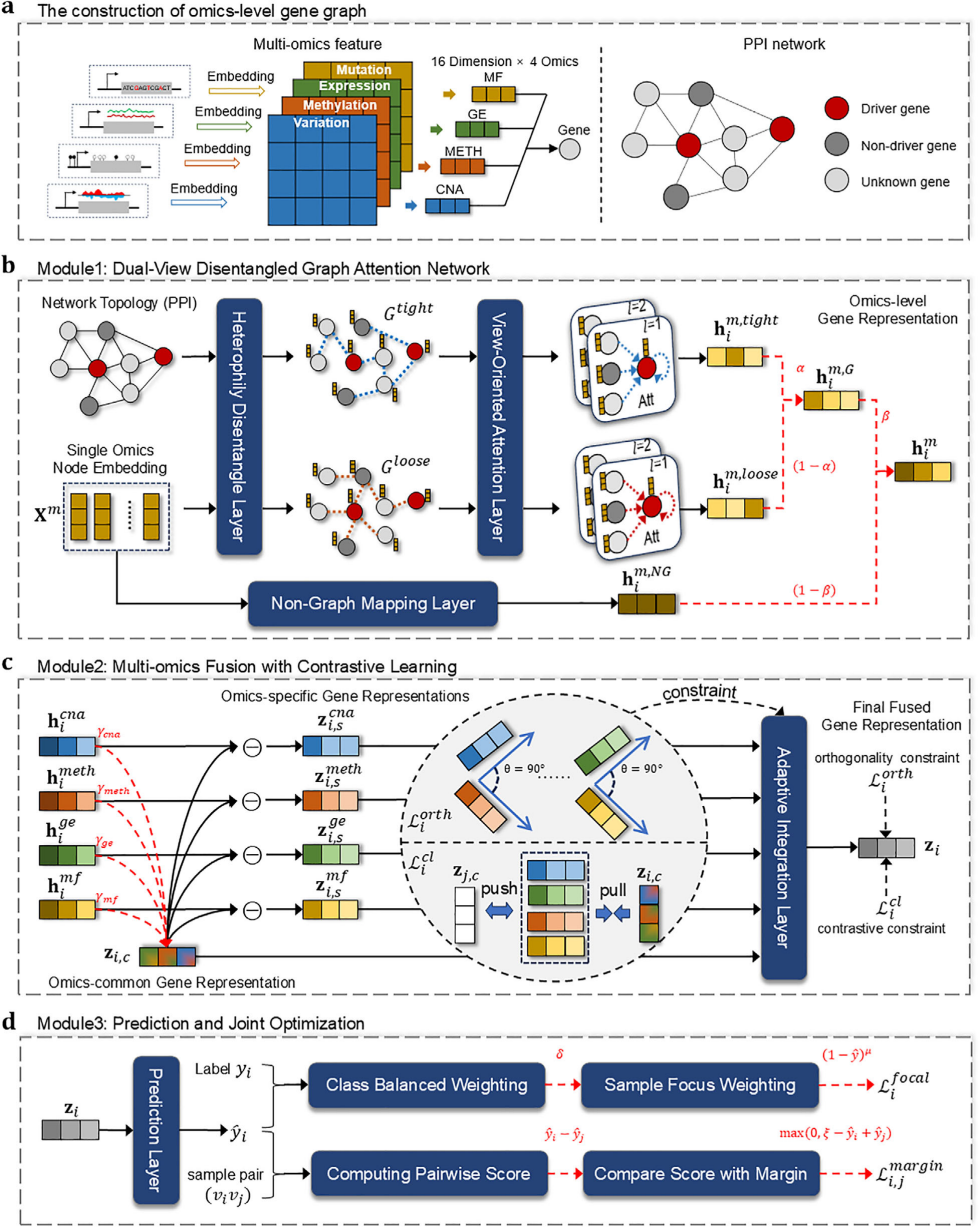
DRIVE framework for disentangled multi‐omics graph learning. (a) MF, METH, GE and CNA profiles are projected separately into latent omics‐level gene embeddings and combined with a shared PPI topology to define omics‐level gene graphs. (b) Within each omics‐level graph, DRIVE decomposes PPI neighborhoods into tight and loose views according to embedding consistency, aggregates each view with view‐oriented attention and integrates propagated graph information with intrinsic omics signals from a non‐graph branch. (c) DRIVE learns gene‐adaptive omics‐common and omics‐specific representations through residual disentanglement, contrastive alignment and orthogonality regularization, then integrates them into a final fused representation. (d) The fused representation is mapped to a driver probability and trained with focal loss, pairwise margin‐ranking loss and self‐supervised representation objectives.

DRIVE is a semi‐supervised graph representation framework that disentangles network context, decomposes multi‐omics evidence, and jointly optimizes representation learning with driver‐gene classification (Figure 1). The input integrates gene labels, pan‐cancer multi‐omics profiles, and PPI networks into omics‐level gene graphs (Figure 1a). Positive labels were collected from NCG v6.0 [9], COSMIC CGC v91 [10] and DigSEE [36], whereas genes without evidence in these resources were used to define stringent negative samples; the remaining genes were treated as unlabeled. For each gene, MF, CNA, METH, and GE were represented across 16 cancer types, yielding a 64‐dimensional feature vector. Six PPI networks, CPDB [37], STRINGdb [38], MULTINET [39], PCNet [40], IRefIndex [41] and IRefIndex2015 [42], provided the graph topologies summarized in Table 1.

**TABLE 1:** Statistics of the six benchmark datasets.

Dataset	#Nodes	#Edges	#Positive	#Negative	#Unlabeled
CPDB	13 627	252 189	796	2187	10 644
IRefIndex	17 013	369 361	836	4056	12 121
IRefIndex2015	12 129	91 809	785	1973	9371
MULTINET	14 398	109 567	790	3709	9899
PCNet	19 780	2 724 723	859	5483	13 438
STRINGdb	13 179	336 548	783	2415	9981

The first module reduces heterophilic signal mixing through a dual‐view disentangled graph attention network (Figure 1b). For each omics‐level graph, DRIVE separates the original PPI neighborhood into a tight view and a loose view according to latent similarity between omics‐level gene embeddings. The tight view emphasizes interactions between genes with concordant molecular profiles, whereas the loose view preserves divergent interactions that may contain complementary biological context. A view‐oriented attention layer then weights the two views, and a parallel non‐graph branch retains intrinsic omics alterations that could otherwise be diluted by propagation. The graph‐derived and intrinsic representations are integrated to produce an omics‐level representation for each gene.

The second module converts heterogeneous molecular evidence into a gene‐adaptive fused representation (Figure 1c). DRIVE first learns an omics‐common representation by weighting the four omics‐level representations for each gene. It then extracts omics‐specific residual components by removing the shared signal from each omics branch. A contrastive objective aligns each omics‐specific representation with the corresponding omics‐common representation for the same gene, while a soft orthogonality constraint discourages redundancy among source‐specific components. An adaptive integration layer combines the common and specific components according to their gene‐specific contributions.

The third module maps the final fused representation to a driver probability and trains DRIVE under sparse labels (Figure 1d). Focal loss increases the contribution of difficult and minority‐class driver samples, and pairwise margin ranking loss encourages annotated drivers to score above annotated non‐drivers. These supervised objectives are optimized jointly with the contrastive and orthogonality objectives, allowing unlabeled genes to contribute to representation learning. After training, genes are ranked by predicted driver probability to nominate high‐confidence candidate driver genes.

### 2.2 Multi‐Omics Sources Contribute in a Network‐Adaptive Manner

We first asked whether each molecular modality contributed useful information to DRIVE. In a conventional two‐layer GCN, simple concatenation of non‐empty combinations of MF, CNA, METH, and GE did not consistently improve prediction and sometimes reduced performance relative to the strongest single‐omics signal (Section S1). This behavior indicates that naive fusion can introduce negative gains when redundant or conflicting modalities are compressed into one representation. We therefore compared full DRIVE with four single‐omics removal variants: DRIVE w/o MF, DRIVE w/o CNA, DRIVE w/o GE and DRIVE w/o METH.

**FIGURE 2:**
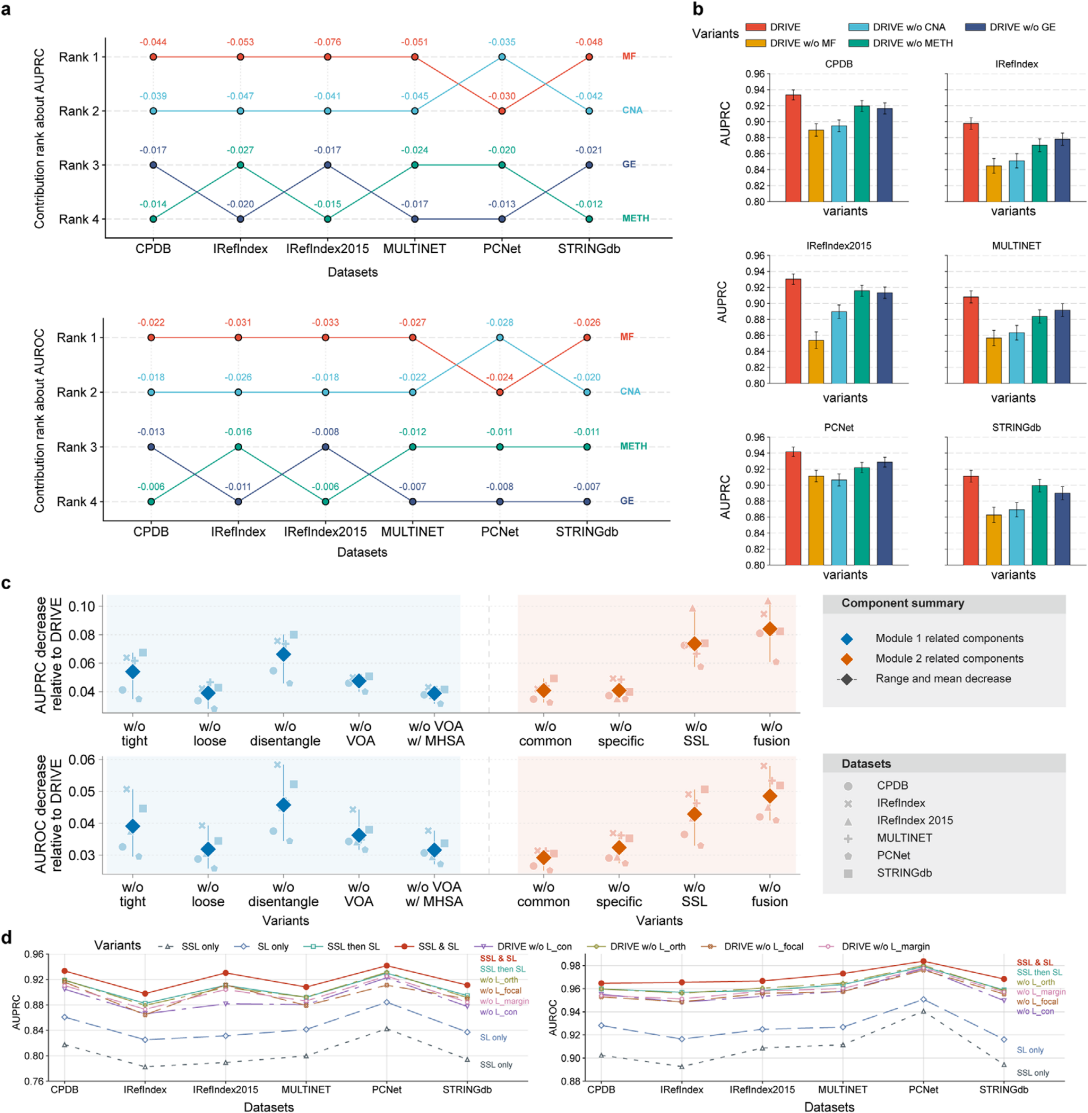
Multi‐omics and module ablations in DRIVE. (a) Contribution ranking of four omics sources estimated from AUPRC and AUROC decreases after removing each source from full DRIVE. Higher ranks indicate larger performance losses and stronger predictive contributions; annotated values report the performance decrease relative to full DRIVE. (b) AUPRC comparison between full DRIVE and single‐omics removal variants across six benchmark PPI networks. Bars show mean test performance and error bars denote standard deviation across fivefold cross‐validation runs. MF, mutation frequency; CNA, copy‐number aberration; METH, DNA methylation; GE, gene expression. (c) Component‐level ablation of Module 1 and Module 2. Performance decrease was calculated as the full DRIVE score minus the ablated score. Points denote individual PPI networks, diamonds denote the mean decrease and vertical bars denote the observed range. (d) AUPRC and AUROC comparison of eight training strategies across the six PPI networks. SSL & SL denotes the joint self‐supervised and supervised learning strategy used in DRIVE. VOA, view‐oriented attention; MHSA, multi‐head self‐attention; SSL, self‐supervised learning; SL, supervised learning.

Full DRIVE achieved a mean AUPRC of 0.9204 and a mean AUROC of 0.9704 across the six PPI networks. Removing any omics source reduced performance (Figure 2a,b and Table S1), showing that all four modalities supplied predictive information. MF provided the strongest and most stable contribution on most networks: removing MF reduced the mean AUPRC to 0.8698, an average decrease of 0.0506, and reduced mean AUROC to 0.9432, a decrease of 0.0272. CNA was the dominant source on PCNet and had the second‐largest average effect overall, reducing mean AUPRC to 0.8791 and mean AUROC to 0.9480. METH and GE produced smaller but consistent gains, reducing mean AUROC by 0.0105 and 0.0095 when removed, respectively. These patterns indicate that DRIVE does not impose a fixed omics hierarchy, but adapts the contribution of each modality to the network context.

We next examined the absolute performance loss caused by removing each omics source (Figure 2b). Although METH and GE contributed less than MF and CNA on average, removing either modality decreased AUPRC on every PPI network. Removing METH reduced mean AUPRC from 0.9204 to 0.9018, and removing GE reduced it to 0.9030. The largest METH effects occurred on IRefIndex and MULTINET, whereas the largest GE effect occurred on STRINGdb. Thus, DRIVE converted each omics source into a positive predictive gain while preserving network‐specific contribution patterns.

### 2.3 Ablation Analyses Identify the Design Choices Driving DRIVE Performance

We next tested which components accounted for the performance gains of DRIVE (Figure 2c,d and Table S2). For Module 1, five variants examined topology‐level disentanglement and view‐specific aggregation: DRIVE w/o tight, DRIVE w/o loose, DRIVE w/o disentangle, DRIVE w/o VOA, and DRIVE w/o VOA w/ MHSA. Removing the full dual‐view disentanglement module reduced mean AUPRC from 0.9204 to 0.8735 and mean AUROC from 0.9704 to 0.9439. Removing the tight view caused a larger loss than removing the loose view, indicating that molecularly concordant neighborhoods provide stronger structural evidence for driver prediction. Removing view‐oriented attention reduced mean AUPRC by 0.0276, and replacing it with standard multi‐head self‐attention still reduced AUPRC by 0.0188. These results support both the tight/loose topology split and the view‐specific aggregation design.

For Module 2, four variants tested common and specific omics decomposition and self‐supervised optimization. Removing the full multi‐omics fusion module produced the largest component‐level loss, reducing mean AUPRC from 0.9204 to 0.8562 and mean AUROC from 0.9704 to 0.9415. Removing the self‐supervised objective also caused a substantial decrease, with mean AUPRC falling to 0.8667 and mean AUROC to 0.9472. Removing either the omics‐common or omics‐specific component produced comparable AUPRC losses of 0.0208. Thus, shared cross‐omics evidence and source‐specific residual signals both contributed to the final representation.

Because driver‐gene discovery relies on sparse labels and large unlabeled gene spaces, we compared four training strategies and four single‐loss ablations for Module 3 (Figure 2d and Table S2). SSL only used the self‐supervised objectives without driver labels; SL only used focal and pairwise ranking losses; SSL then SL used sequential pretraining and fine‐tuning; and SSL & SL jointly optimized the two objective classes. Joint SSL & SL achieved the best mean AUPRC and AUROC, 0.9204 and 0.9704, across all networks. SSL only performed worst (mean AUPRC 0.8043; mean AUROC 0.9084), confirming that representation learning alone is insufficient for class discrimination. SL only improved performance but remained below the joint strategy, whereas SSL then SL approached full DRIVE but still underperformed it by 0.0157 AUPRC and 0.0076 AUROC. Simultaneous optimization therefore aligned the representation space with the driver‐prioritization objective more effectively than sequential training. We further removed each loss term individually. Removing *L*_con_, *L*_orth_, *L*_focal_, and *L*_margin_ reduced mean AUPRC to 0.8889, 0.9034, 0.8952, and 0.8972, respectively, with corresponding AUROC values of 0.9570, 0.9633, 0.9578, and 0.9597. These results show that contrastive alignment provides the strongest individual contribution, while orthogonality regularization, imbalance‐aware classification, and pairwise ranking provide complementary gains within the joint optimization framework.

DRIVE was also stable to the key hyperparameters governing topology construction and joint representation learning (Section S2). Across the six PPI networks, the best overall performance was obtained at τ  =  0.5, *t*  =  0.5, and η  =  0.3 (Figure S1). Training and validation losses decreased toward stable plateaus, accompanied by convergent validation performance (Figures. S2 and S3). Meanwhile, inter epoch edge reassignment progressively declined during optimization, indicating stabilization of the dynamically constructed graph views (Figure S4).

### 2.4 DRIVE Disentangles Heterophilic Topology and Multi‐Omics Representations

**FIGURE 3:**
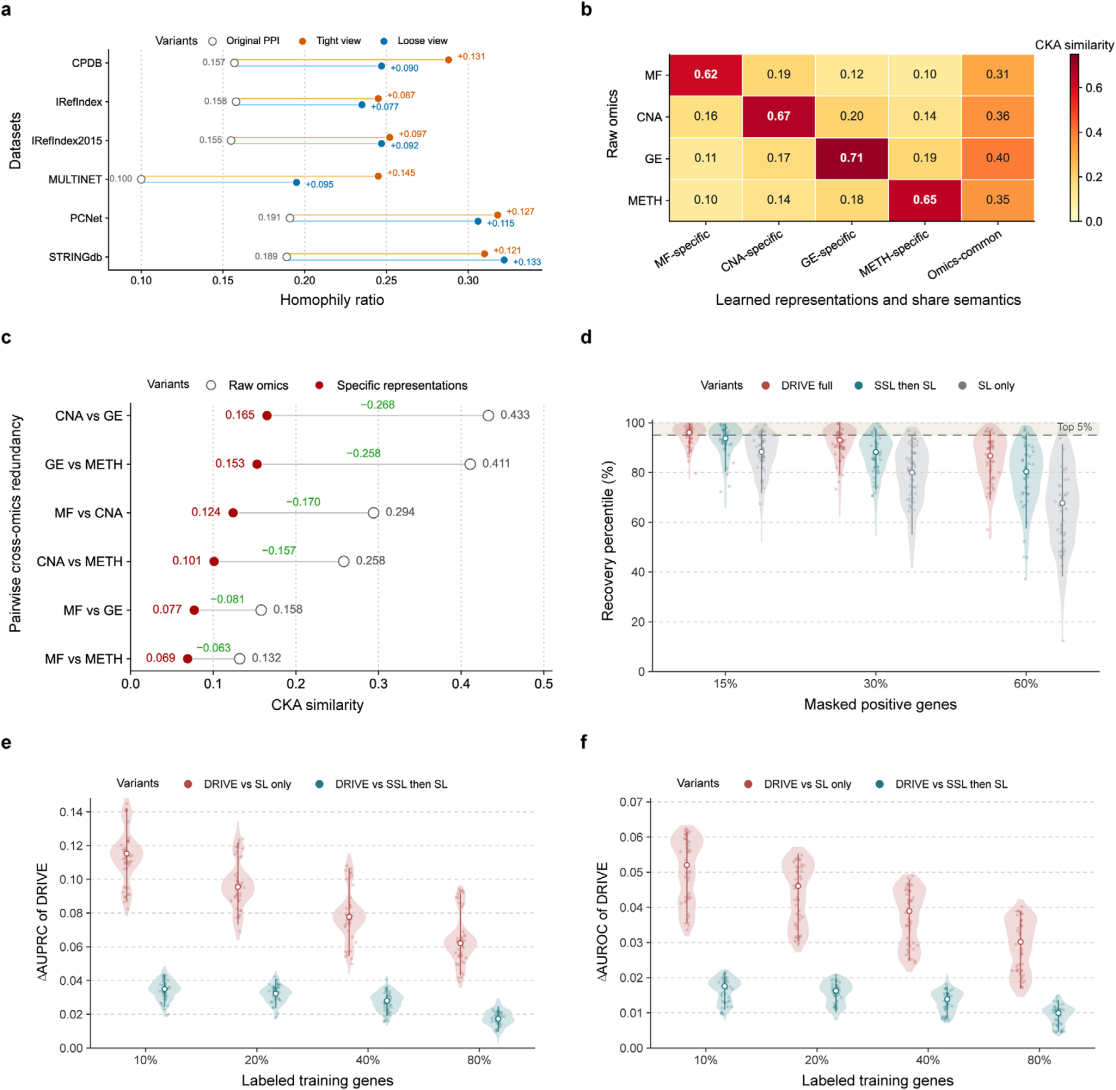
Topology and representation disentanglement under sparse annotations. (a) One‐hop node homophily ratios for the original PPI networks and the DRIVE‐learned tight and loose views. Annotated values indicate the homophily increase relative to the original PPI network. (b) CKA similarity between raw omics features and learned representations, averaged across the six PPI networks. Diagonal enrichment indicates preservation of source‐specific information, whereas the common representation captures balanced cross‐omics semantics. (c) Pairwise CKA similarity among raw omics features and among learned omics‐specific representations. Lower CKA after disentanglement indicates reduced cross‐omics redundancy. (d) Recovery of masked KDGs under different training strategies. For each masking ratio, 60 masked driver genes were uniformly sampled from the hidden positive set; each point denotes one sampled masked KDG, and the dashed line marks the top 5% threshold. (e,f) Label‐ratio sensitivity measured as AUPRC (e) and AUROC (f) gains over comparison training strategies. For each label ratio, each PPI network was evaluated with fivefold cross‐validation and 10 random seeds per fold. KDG, known driver gene; SSL, self‐supervised learning; SL, supervised learning.

We examined how Module 1 reshaped heterophilic PPI topology into more label‐consistent semantic views. The original one‐hop homophily ratios were low across benchmark networks, ranging from 0.1002 on MULTINET to 0.1912 on PCNet, indicating that direct neighborhood aggregation can mix genes with discordant driver status (Table S3). After dual‐view disentanglement, both learned graph views showed increased homophily on all networks (Figure 3a). The tight view increased homophily by 0.087 to 0.145, and the loose view by 0.077 to 0.133. On average, DRIVE increased the original homophily ratio of 0.158 by 0.118 in the tight view and 0.100 in the loose view. These results show that DRIVE reorganizes heterophilic PPI neighborhoods into more label‐consistent contexts while retaining complementary divergent interactions.

We then used centered kernel alignment (CKA) to test whether Module 2 preserved source‐specific evidence while extracting an omics‐common representation. Each omics‐specific representation retained the highest similarity to its corresponding raw omics source (Figure 3b): MF‐specific, CNA‐specific, GE‐specific and METH‐specific representations had CKA values of 0.62, 0.67, 0.71, and 0.65 with their matched inputs, respectively. Similarities to non‐corresponding omics sources were mostly below 0.20. By contrast, the omics‐common representation showed a balanced similarity pattern across MF, CNA, GE and METH (0.31, 0.36, 0.40, and 0.35). This diagonal enrichment together with the balanced common component supports the intended separation between source‐specific and shared multi‐omics evidence.

Finally, we quantified cross‐omics redundancy before and after representation learning (Figure 3c). Raw omics features showed substantial pairwise similarity, especially CNA versus GE (CKA = 0.443) and GE versus METH (CKA = 0.411). After DRIVE learned omics‐specific representations, these similarities decreased to 0.165 and 0.153, respectively. The same trend was observed for MF versus CNA, CNA versus METH, MF versus GE and MF versus METH. Overall, mean pairwise CKA decreased from 0.283 in raw omics features to 0.115 in omics‐specific representations. This reduction in redundancy explains why removing the fusion module produced the largest component‐level performance loss.

### 2.5 DRIVE Prioritizes Hidden Driver Genes under Sparse Annotations

We next evaluated whether DRIVE could recover hidden driver genes from a largely unlabeled gene space. Known driver genes were masked during training and then ranked by the trained model during prediction. A higher recovery percentile indicates that the masked driver gene was placed closer to the top of the predicted candidate list. Full DRIVE recovered masked driver genes at higher percentiles than SSL then SL and SL only across all masking ratios (Figure 3d). When 15% of known drivers were masked, DRIVE recovery distributions concentrated near the top 5% threshold, whereas the alternative strategies produced broader and lower ranking distributions. Under 30% and 60% masking, DRIVE maintained higher recovery percentiles, showing that joint optimization improves candidate prioritization even when a large fraction of positive labels is hidden.

We further reduced the total annotation budget by training with 10%, 20%, 40% or 80% of labeled genes and measuring AUPRC gains over SL only and SSL then SL (Figure 3e). DRIVE maintained positive AUPRC gains at every label ratio. The advantage over SL only was largest under the most limited supervision, with average gains of approximately 0.11 at the 10% label ratio and 0.09 at the 20% label ratio. The gain decreased as more labels became available but remained positive at 40% and 80%. AUROC showed the same pattern (Figure 3f). DRIVE improved over SL only and SSL then SL at every label ratio, with the largest AUROC gain over SL only, approximately 0.05, at the 10% label ratio. The joint SSL & SL variant achieved higher mean AUPRC and AUROC than SSL then SL, SL only and SSL only.

We further tested robustness to incomplete PPI topology by randomly removing 5%, 10% or 20% of interactions from each benchmark network before training (Section S3 and Figure S5). Performance decreased gradually with increasing edge removal but remained stable under substantial structural perturbation. Even after removing 20% of PPI edges, mean AUPRC decreased only from 0.9204 to 0.9028 and mean AUROC from 0.9704 to 0.9626. The degradation was smallest on the dense PCNet network and more pronounced on the relatively sparse IRefIndex2015 and MULTINET networks, indicating network‐dependent sensitivity to structural incompleteness.

We also evaluated sensitivity to negative sample construction using a CancerMine filtered negative set and a randomly reduced 50% negative set (Section S4 and Figure S6). Compared with the original setting, mean AUPRC changed from 0.9204 to 0.9234 and 0.9171, respectively, while mean AUROC changed from 0.9704 to 0.9720 and 0.9689. These small variations indicate that DRIVE remains stable under both stricter negative annotation and substantially reduced negative supervision.

### 2.6 DRIVE Outperforms Existing Methods Across Diverse PPI Networks

We compared DRIVE with ten representative driver‐gene prediction methods, GCN [25], EMOGI [27], SMG [35], SGCD [30], deepCDG [31], MFC‐GCN [43], MODCAN [33], GRAFT [28], TREE [29] and MNDGNN [44](Section S5). These baselines cover conventional graph convolution, explainable graph learning, self‐supervised graph learning, heterophily‐aware representation learning, multi‐omics fusion, multi‐network fusion and Transformer‐based graph modeling. All methods were evaluated on CPDB, STRINGdb, MULTINET, PCNet, IRefIndex and IRefIndex2015 using identical multi omics inputs, data splits and evaluation metrics under the same semi supervised fivefold cross validation protocol.

**FIGURE 4:**
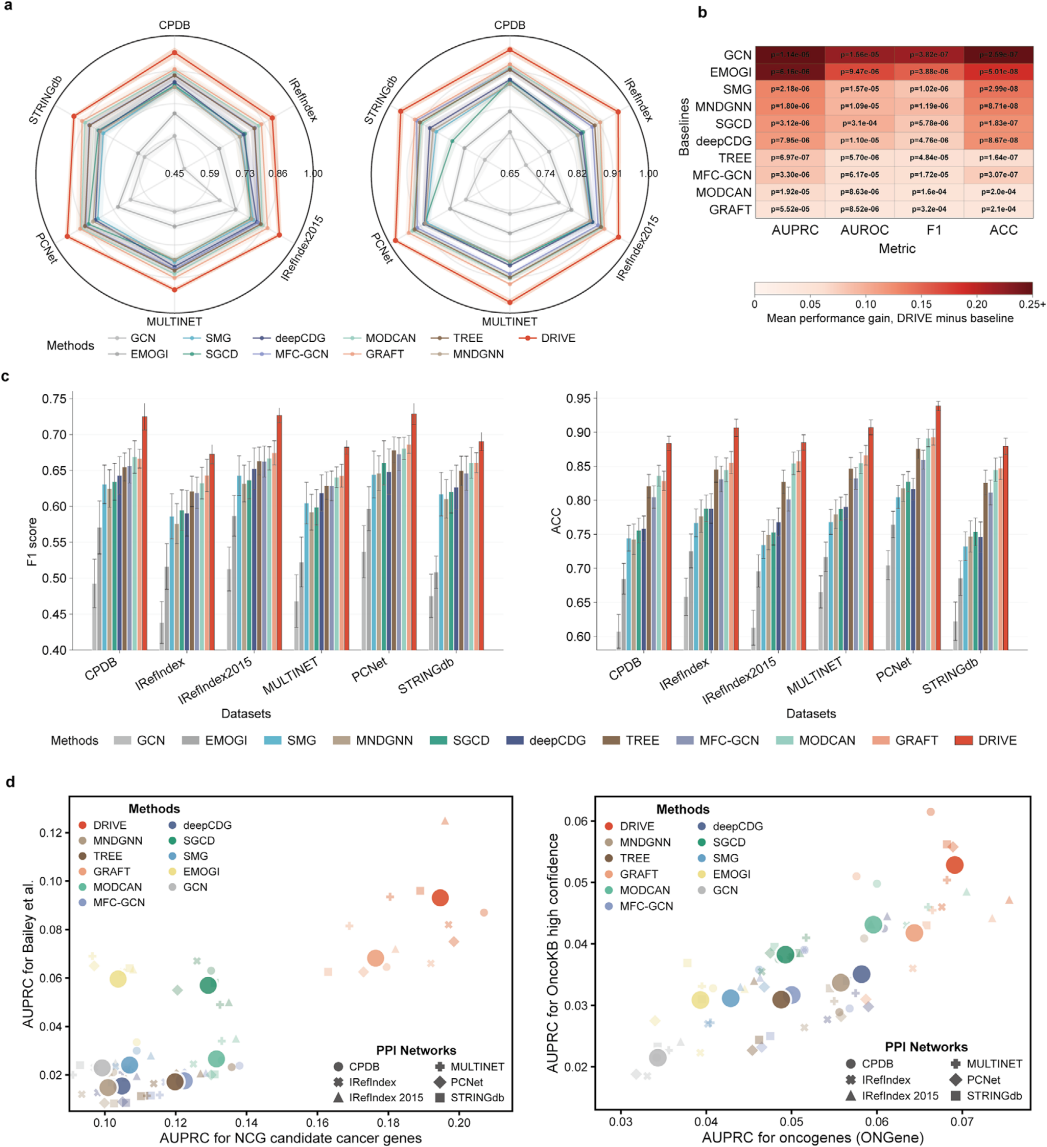
Benchmark and external validation performance of DRIVE. (a) AUPRC and AUROC comparison of DRIVE with ten baseline methods across CPDB, IRefIndex, IRefIndex2015, MULTINET, PCNet and STRINGdb. Values denote mean test performance from fivefold cross‐validation; outer radar profiles indicate higher performance. (b) Statistical comparison between DRIVE and each baseline across AUPRC, AUROC, F1 and ACC. Cell values denote *P* values from two‐sided paired t tests across the six PPI networks, and color intensity denotes the mean performance gain of DRIVE over the corresponding baseline. (c) F1 and ACC comparison across the six PPI networks. Bars show mean test performance and error bars denote standard deviation across cross‐validation runs. (d) External validation using external cancer‐gene resources. Small points denote method performance on individual PPI networks, point shapes denote PPI networks, colors denote methods and large circles denote mean performance across networks. Higher AUPRC indicates stronger recovery of external cancer‐gene sets.

Because cancer driver labels are highly imbalanced, AUPRC was used as the primary metric. DRIVE achieved the highest AUPRC on all six PPI networks and formed the outermost radar profile (Figure 4a). Its mean AUPRC was 0.9204, compared with 0.8680 for GRAFT, the strongest competing method on average. The advantage persisted on both sparse and dense networks, including IRefIndex2015 (AUPRC 0.9303) and PCNet (AUPRC 0.9417). Relative to the strongest baseline on each dataset, DRIVE improved AUPRC by 4.26% to 7.66%. AUROC showed the same trend, with DRIVE reaching a mean of 0.9704 compared with 0.9277 for GRAFT and improving over the strongest baseline by 3.95% to 5.74%.

We assessed whether these gains were consistent across networks using two‐sided paired t tests (Figure 4b). DRIVE improved over all baselines across AUPRC, AUROC, F1 and ACC, including comparisons with the strongest recent methods (Table S4). The advantage was also evident in threshold‐dependent metrics (Figure 4c). DRIVE achieved the highest mean F1 score (0.7042) and ACC (0.9001), compared with 0.6623 and 0.8580 for GRAFT and 0.6582 and 0.8544 for MODCAN. Thus, DRIVE improved not only ranking quality under class imbalance but also binary driver‐gene classification.

The ordering of the baseline methods further supports the design rationale of DRIVE. GCN and EMOGI, which propagate multi‐omics features over the original PPI topology, showed the weakest performance, consistent with the expected cost of heterophilic signal mixing. SMG improved over EMOGI, indicating the value of self‐supervised graph learning under sparse annotations. SGCD and deepCDG improved further by adding heterophily‐aware representation separation and multi‐omics fusion, respectively, while MFC‐GCN, MODCAN and GRAFT benefited from stronger graph and fusion designs. TREE introduced Transformer based structural representation learning, while MNDGNN incorporated directed graph modeling originally developed for multiplex biological networks. DRIVE outperformed these approaches by addressing heterophilic topology, heterogeneous omics evidence and sparse supervision within one framework.

We then tested generalization beyond the curated training labels using four external cancer‐gene resources: OncoKB [45], ONGene [46], NCG candidate cancer genes [9] and the Bailey et al. driver‐gene set [47] (Figure 4d). To ensure independence, positive samples that overlap with the training set in these external resources have been removed (Section S6 and Table S5). DRIVE achieved the highest mean AUPRC on all four resources, with values of 0.0529 on OncoKB, 0.0691 on ONGene, 0.1947 on NCG and 0.0931 on Bailey et al. The strongest baselines reached 0.0430, 0.0646, 0.1766 and 0.0720, respectively. Although the absolute metric is low due to the insufficient number of true positive samples available after removing overlaps, we can still observe the advantages of DRIVE through the relative metric. Across 24 network‐resource combinations, DRIVE ranked first in 23, with the only exception nearly tied with the best baseline. These results indicate that DRIVE captures transferable cancer‐related signals beyond the specific labels used for training.

We further examined the generalization of DRIVE across different biological network structures and cancer contexts (Section S7 and Figures S7 and S9). DRIVE achieved the best AUPRC and AUROC on all five single‐network settings, including protein complex, KEGG pathway, RegNetwork, DawnNet, and kinase‐substrate. In the multi‐network setting, an attention‐based DRIVE integration strategy outperformed both the original MNDGNN and TREE models as well as simpler DRIVE fusion variants, indicating that DRIVE's potential for future multi‐network expansion. We further evaluated six cancer‐specific datasets, BRCA, COAD, LUAD, LIHC, BLCA, and THCA. DRIVE achieved the best performance across all six cancer contexts, supporting its generalization under different network topologies and cancer backgrounds.

To enhance the interpretability of DRIVE, we employ GNNExplainer [48] on the well‐trained model (Section S8 and Figure S10). Representative candidate genes showed distinct combinations of molecular and network evidence: RELA was dominated by GE and MF attribution together with an NF κB associated local neighborhood, whereas FYN showed stronger MF and GE attribution with signaling related PPI neighbors. Across the PCDG set, global omics attribution was highest for MF and CNA, while gene specific explanations remained heterogeneous across all four omics sources. Within graph derived attribution, tight views accounted for 68.0% and loose views for 32.0%, supporting the complementary structural roles of the two learned graph views.

### 2.7 DRIVE Candidate Drivers are Supported by Network Proximity and CRISPR Dependency

**FIGURE 5:**
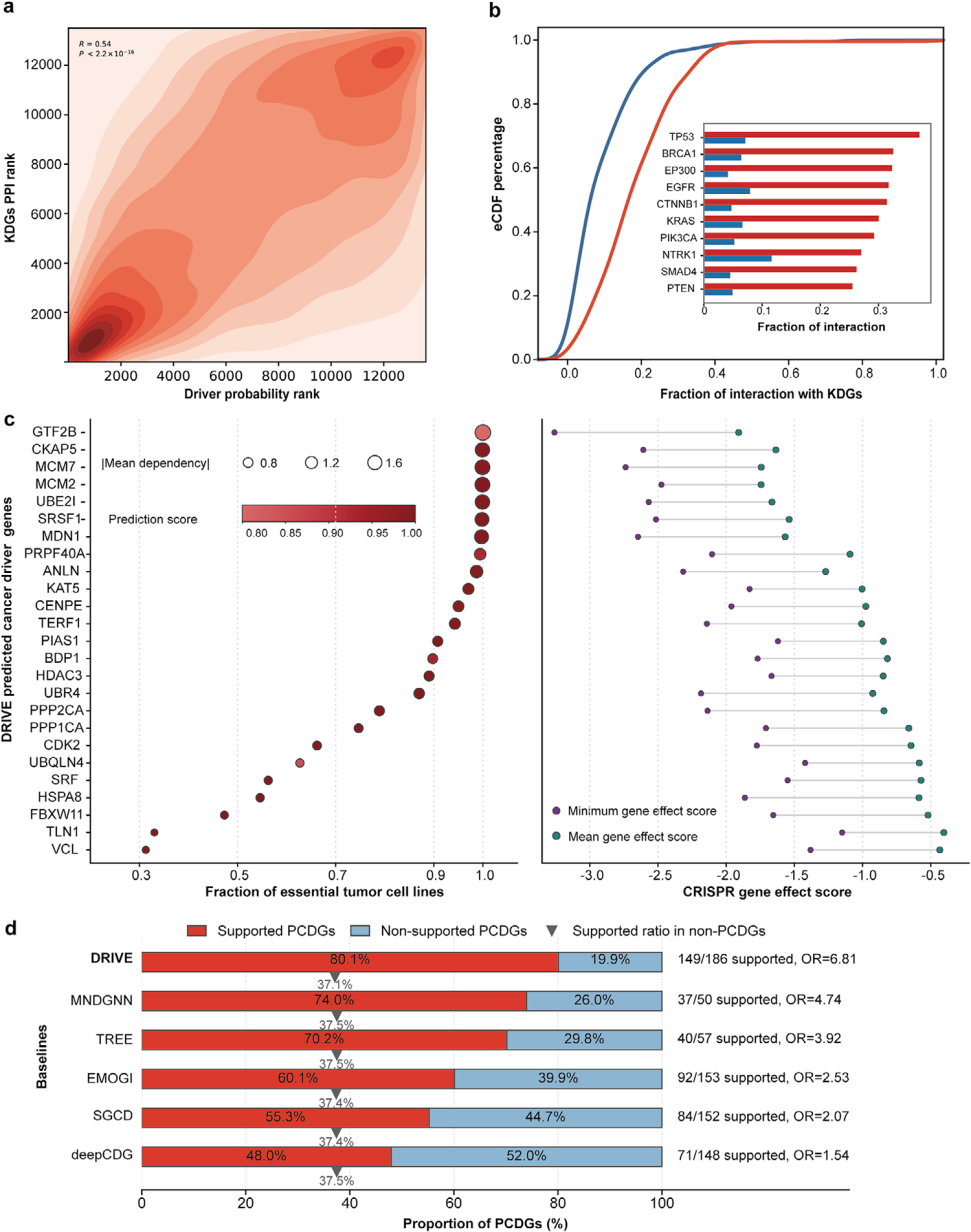
Network and CRISPR support for DRIVE candidate driver genes. (a) Two‐dimensional density plot showing the rank correlation between DRIVE driver probability and KDG interaction rank across all genes. Color density denotes the local concentration of genes in rank space. (b) Empirical cumulative distribution functions comparing PCDGs and background genes by the fraction of PPI neighbors that are KDGs. The inset shows the top ten KDGs most frequently interacting with PCDGs; red bars indicate the fraction of PCDGs directly interacting with each KDG, and blue bars indicate the corresponding fraction among all network genes. (c) CRISPR dependency profiles of PCDGs across DepMap tumor cell lines. The left panel shows the fraction of cell lines in which each gene is essential (gene‐effect score ≤ −0.50), with point color denoting driver probability and point size denoting absolute mean dependency. The right panel summarizes minimum and mean gene‐effect scores; lower scores indicate stronger growth inhibition after perturbation. (d) CRISPR dependency support among PCDG lists predicted by DRIVE, EMOGI, SGCD, deepCDG, TREE and MNDGNN. Stacked bars show supported and non‐supported proportions; gray triangles indicate supported ratios among non‐PCDGs, and text reports supported counts, odds ratios and Fisher exact test *P* values. KDG, known driver gene; PCDG, predicted candidate driver gene.

We next evaluated whether genes prioritized by DRIVE showed biological support beyond benchmark labels. First, we compared DRIVE driver‐probability ranks with PPI proximity to known driver genes (KDGs). Each gene was assigned a driver‐probability rank and a KDG‐interaction rank based on the number of one‐hop PPI interactions with KDGs. The two ranks were positively correlated across all genes (*R* = 0.54, *P* < 2.2 × 10^−16^; Figure 5a), indicating that high‐priority DRIVE genes tended to occupy KDG‐enriched network neighborhoods. By projecting PPI topology and complex interactions among pan‐cancer molecular features into a unified embedding space, DRIVE is able to identify not only candidates with numerous known driver genes as first‐order neighbors (e.g., RELA, NFKB1) but also those with fewer direct associations to known driver genes (e.g., HERC2, UBR4).

We then focused on predicted candidate driver genes (PCDGs). The top 100 high‐confidence genes predicted by DRIVE across the six PPI networks were collected and annotated KDGs were removed, yielding 186 PCDGs (Table S6). Empirical cumulative distribution analysis showed that PCDGs had higher KDG‐neighbor fractions than background genes (*P* = 2.4 × 10^−3^, two‐sided test; Figure 5b). The inset analysis further showed enrichment around KDGs frequently connected to PCDGs, including TP53, BRCA1 and EP300.

Functional dependency evidence from DepMap CRISPR screens provided an independent test of candidate relevance [49, 50]. Using CRISPR gene‐effect scores from 1,086 tumor cell lines, a dependency event was defined as a gene‐effect score ≤ −0.50. Representative PCDGs showed broad dependency across tumor cell lines and strong negative gene‐effect scores (Figure 5c). Examples included GTF2B, which is involved in basal transcription [51]; CKAP5, which supports microtubule dynamics, spindle function and chromosome segregation [52]; and MCM7 and MCM2, which are components of the MCM helicase complex [53].

We compared DRIVE with EMOGI, SGCD, deepCDG, TREE, and MNDGNN using the same DepMap support criterion. A candidate gene was classified as CRISPR‐dependency supported if dependency events occurred in at least 10% of the 1,086 tumor cell lines. DRIVE had the highest supported proportion, with 149 of 186 PCDGs supported (80.1%; OR = 6.81; *P* = 1.0 × 10^−2^, Fisher exact test; Figure 5d). The supported proportions were 74.0% for MNDGNN, 70.2% for TREE, 60.1% for EMOGI, 55.3% for SGCD, and 48.0% for deepCDG. Compared to these methods, the unique PCDGs identified by DRIVE are also supported by biological evidence (Section S9). For example, the DRIVE‐specific candidate PPP2CA showed broad cancer cell dependency. Thus, DRIVE identified candidate genes with stronger functional dependency support than existing methods under the same evaluation criterion.

According to OncoKB, ONGene, NCG candidate cancer genes, and Bailey et al., 105 PCDGs (56.5%) were previously reported, whereas 81 (43.5%) were provisionally unreported (Section S10 and Figure S11). Direct comparison with nonoverlapping NCG v6.0 known cancer genes showed that the unreported PCDGs retained substantial STRING v12.0 network proximity to known cancer genes, comparable DepMap 23Q4 CRISPR dependency breadth and TCGA PanCancer MC3 somatic alteration patterns, including maximum mutation‐positive patient fractions across 16 cancer types, and comparable recurrence after accounting for background mutability (Section S10 and Figure S12). Driver associated genomic analyses across all 186 PCDGs further showed positive selection (*dN/dS* = 1.104, *P* = 1.73 × 10^−^^8^), recurrence above mutability matched expectations in 13 of 16 cancer types, providing cancer type specific recurrence evidence after background mutability adjustment, and recurrent hotspot enrichment (OR = 3.698, *P* = 0.00218). Among the 81 provisionally unreported candidates, 11 genes were supported by at least two of three independent genomic layers: TCGA mutability matched recurrence, replication in an independent patient cohort, and approximate clonality, providing focused candidates for subsequent functional validation (Section S11 and Figure S13).

### 2.8 DRIVE Links Candidate Drivers to Immune Regulation and Drug Sensitivity

We next investigated whether DRIVE‐predicted candidate driver genes (PCDGs) were associated with tumor immune regulation and therapeutic response. Pathway‐level analyses were performed using the complete prespecified PCDG set listed in Table S6. The Top 20 genes in the pathway‐support map and representative genes in the immune network were ranked using the ImmunePriorityScore (Section S12 and Table S7). We further evaluated the robustness of these findings using an immune‐agnostic selection strategy based solely on DRIVE prediction probabilities, together with random‐gene‐set permutation controls (Section S13 and Figures S14 and S15).

**FIGURE 6:**
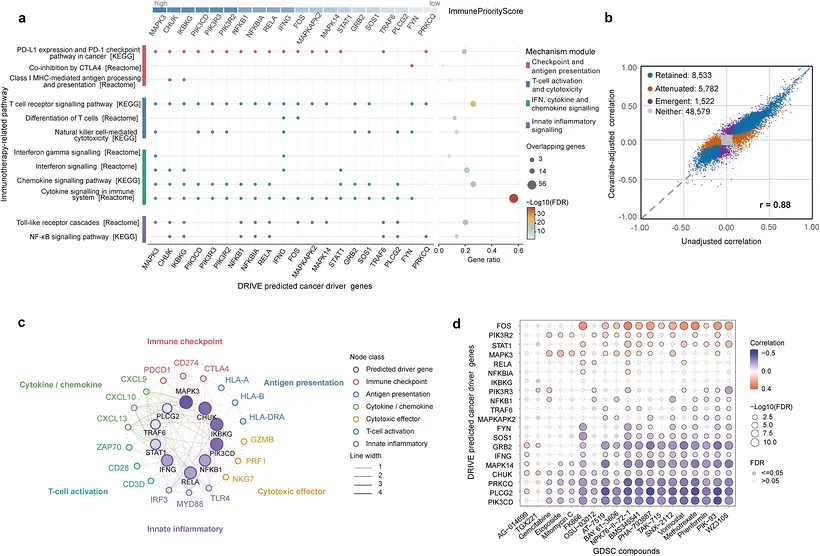
Immune and drug‐response associations of DRIVE candidate driver genes. (a) Immunotherapy‐related pathway support map for the top 20 PCDGs ranked by ImmunePriorityScore. Rows denote curated KEGG and Reactome pathways, and columns denote PCDGs ordered by ImmunePriorityScore. Dots indicate pathway membership. The enrichment summary reports pathway‐level enrichment among PCDGs, with gene ratio on the *x*‐axis, dot size indicating overlapping gene count and dot color indicating FDR‐adjusted significance; pathways with FDR < 0.05 were considered significant. (b) Comparison of unadjusted correlations and covariate adjusted partial correlations across all evaluable gene and immune cell associations. Associations are classified as retained, attenuated, emergent, or nonsignificant under both models. The dashed diagonal indicates equal unadjusted and adjusted effect sizes. Pearson *R* = 0.880. (c) Immune‐regulatory network of PCDGs. Predicted driver nodes are the top ten PCDGs ranked by immune priority. Node color denotes functional class, and edge width indicates the number of shared immune pathway or module supports between connected nodes. (d) Association between PCDG expression and GDSC drug sensitivity from Gene Set Cancer Analysis. Rows denote PCDGs and columns denote drugs. Dot color indicates the correlation between mRNA expression and drug response, dot size indicates FDR, black outlines denote FDR ≤ 0.05 and gray outlines denote FDR > 0.05. PCDG, predicted candidate driver gene.

We examined the enrichment of PCDGs across immunotherapy‐related pathways curated from KEGG [54] and Reactome [55] and organized into five functional categories: immune checkpoint and antigen presentation, T‐cell activation and cytotoxicity, interferon signaling, cytokine and chemokine signaling, and innate inflammatory signaling. Using all pathway‐annotated non‐KDGs as the background, enrichment was assessed by Fisher's exact test followed by Benjamini‐Hochberg correction, with FDR < 0.05 considered significant (Figure 6a). PCDGs were significantly enriched in multiple immune programs, with the strongest signals observed for cytokine signaling in the immune system (*P* = 2.3 × 10^−9^, FDR = 7.1 × 10^−8^), PD‐L1 expression and PD‐1 checkpoint signaling in cancer (*P* = 1.9 × 10^−4^, FDR = 3.7 × 10^−3^), and T‐cell receptor signaling (*P* = 4.8 × 10^−4^, FDR = 1.5 × 10^−2^). Several high‐priority PCDGs, including MAPK3, CHUK, IKBKG, PIK3CD, PIK3R2, PIK3R3, and NFKB1, were supported across multiple immune functional modules. Importantly, the major pathway signals remained detectable when representative genes were selected solely according to DRIVE prediction probability and significantly exceeded those observed in random subsets of the same PCDG pool (Section S13 and Figures S14 and S15), indicating that these patterns were not dependent on immune‐guided gene prioritization.

We next assessed whether PCDG expression was associated with immune‐cell infiltration across 16 TCGA cancer types. Cancer‐type‐specific models were adjusted for potential confounders, including tumor purity, tumor mutational burden, copy‐number variation, batch effects, and expression collinearity. Across all evaluable PCDG—LM22 immune‐cell pairs, 8533 associations remained significant after covariate adjustment, and adjusted effect sizes were highly concordant with their unadjusted estimates (Pearson *r* = 0.880; Figure 6b). These associations nevertheless showed substantial cancer‐type specificity, indicating that the relationship between individual PCDGs and immune‐cell states varies across tumor contexts.

We further examined the clinical relevance of these immune associations in independent, cancer‐type‐matched immunotherapy cohorts (Table S8). Across cohorts, the direction of association with clinical benefit was consistently positive, yielding a pooled standardized mean difference of 0.50 SD (95% CI, 0.24 to 0.76; *P* = 1.50 × 10^−^^4^; *I^2^* = 32%). Complementary multivariable survival analyses across TCGA cancer types identified additional FDR‐significant associations between PCDG‐related immune features and overall survival (Section S14 and Figures S16–S18). Together, these analyses extend the observed immune associations beyond pathway annotation and support their relevance across tumor immune states, immunotherapy response, and clinical outcome.

Consistent with these robust immune associations, the immune‐regulatory network placed top‐ranked immune‐related PCDGs within coherent signaling contexts (Figure 6c). The NF‐κB‐related region containing CHUK, IKBKG, NFKB1 and RELA connected to immune‐checkpoint nodes, including CD274, PDCD1, and CTLA4, and to chemokines such as CXCL9, CXCL10 and CXCL13. PIK3CD, and PLCG2 connected T‐cell activation and cytotoxic effector modules, whereas IFNG and STAT1 were located near interferon signaling and antigen‐presentation regions. These patterns suggest that DRIVE PCDGs may span multiple immune functional modules involved in tumor immune regulation.

Finally, we investigated the pharmacogenomic relevance of PCDGs using GDSC drug‐response profiles obtained through Gene Set Cancer Analysis [56, 57]^.^ Drug response was represented by IC50, with negative PCDG expression correlations indicating greater drug sensitivity and positive correlations indicating reduced sensitivity. PCDGs were associated with multiple drugs (Figure 6d), including compounds connected to inflammatory or immune‐related mechanisms. For example, FK866 inhibits NAMPT‐mediated NAD+ biosynthesis and has been linked to reduced inflammatory cytokine and chemokine secretion [58], whereas BMS‐345541 inhibits IκB kinase and NF‐κB activation [59]. Together, these analyses suggest that DRIVE‐derived PCDGs provide candidates for follow‐up studies of tumor immune states and therapeutic vulnerabilities.

## 3 Discussion

DRIVE addresses a central obstacle in computational cancer genomics: how to prioritize driver genes when molecular evidence is heterogeneous, biological networks are not label‐homophilic, and curated annotations are incomplete. Rather than treating these issues separately, DRIVE integrates them into one representation‐learning framework. It constructs omics‐level gene graphs, separates PPI neighborhoods into tight and loose semantic views, decomposes molecular evidence into omics‐common and omics‐specific components, and trains the resulting representation with both self‐supervised and driver‐specific objectives. This design links the statistical task of driver‐gene prediction to biological structure in the data.

The first contribution is explicit modeling of heterophilic PPI topology. In protein‐interaction networks, an edge denotes a functional or physical association rather than shared driver status. Direct propagation can therefore pass discordant information between driver and non‐driver genes. DRIVE reduces this problem by learning tight and loose neighborhood views from omics‐level embedding consistency and aggregating them through view‐oriented attention. The increased homophily of both learned views, together with the performance loss caused by removing topology disentanglement, supports the view that biologically meaningful graph propagation requires relation‐aware neighborhood organization rather than unfiltered PPI aggregation.

The second contribution is adaptive multi‐omics fusion. MF and CNA provided dominant predictive evidence in most networks, but METH and GE contributed consistent additional gains. This pattern reflects a broader biological principle: driver activity can be encoded by genomic alteration, gene dosage, epigenetic regulation, and transcriptional state, but these modalities are not interchangeable. DRIVE preserves this distinction by separating a shared omics‐common representation from source‐specific residual components. The CKA analyses show that source‐specific components retained their matched raw modalities while reducing cross‐omics redundancy, explaining why the fusion module was the most influential component in the ablation analysis.

The third contribution is joint learning under sparse supervision. Cancer driver catalogs are incomplete and biased toward well‐studied genes, making purely supervised learning vulnerable to label scarcity and class imbalance. DRIVE combines focal loss and pairwise ranking loss with contrastive and orthogonality objectives, allowing labeled genes to define the prediction task while unlabeled genes shape the representation space. The masked‐driver recovery and label‐ratio experiments show that this joint strategy improves not only benchmark metrics but also the ranking behavior most relevant for discovering missed driver genes.

The performance comparisons indicate that these design choices translate into robust prediction across network resources. DRIVE achieved the highest AUPRC and AUROC on all six PPI networks and improved F1 and ACC as well as ranking metrics. This consistency is important because PPI resources differ substantially in density, coverage and interaction evidence. External validation against OncoKB, ONGene, NCG candidate genes, and the Bailey et al. driver set further suggests that the learned representation captures cancer‐relevant signals beyond the curated labels used for training.

The biological analyses provide a second layer of validation. DRIVE PCDGs were enriched near KDGs in PPI networks and were more often supported by DepMap CRISPR dependency profiles than candidates from EMOGI, SGCD or deepCDG. This finding is important because cross‐validation alone can reward recovery of annotation patterns without demonstrating functional relevance. The CRISPR analysis shows that many DRIVE candidates are linked to cancer‐cell fitness, providing a functional rationale for experimental prioritization.

DRIVE also nominated candidates connected to immune regulation and drug response. Several high‐priority PCDGs mapped to NF‐κB signaling, T‐cell activation, interferon signaling, checkpoint regulation and cytokine or chemokine pathways. Associations with GDSC drug sensitivity further suggest that some candidates may mark pharmacological vulnerabilities. These analyses do not establish causal driver roles, but they show that DRIVE predictions are enriched for genes embedded in interpretable tumor‐immune and therapeutic contexts.

Several limitations define the scope of the present study. DRIVE was evaluated using pan‐cancer multi‐omics summaries and existing PPI resources, so cancer‐type‐specific regulatory networks, single‐cell states, spatial context and dynamic pathway activity remain outside the current model. In addition, computational prioritization and CRISPR dependency support do not replace experimental validation of individual genes in defined tumor models. Future work may extend DRIVE toward more context‐resolved molecular settings. The current dual‐view topology learning could be adapted to cell‐state‐specific or spatially resolved gene relationships, enabling graph representations to capture context‐dependent interactions beyond a static pan‐cancer PPI network. Spatial transcriptomic information could further be incorporated as complementary graph relations to jointly characterize molecular and spatial organization. In parallel, the common‐specific representation scheme may provide a basis for separating transferable pan‐cancer signals from cancer‐type‐specific molecular programs. Combined with self‐supervised learning on unlabeled genes, these extensions could improve knowledge transfer across cancer types with different annotation densities and broaden DRIVE toward single‐cell, spatial, and cancer‐specific driver prioritization.

## 4 Methods

### 4.1 Data Collection and Preprocessing

We constructed six benchmark datasets by integrating gene labels, pan‐cancer multi‐omics profiles, and PPI networks. Positive genes, referred to as known driver genes, were collected from NCG v6.0 [9], COSMIC Cancer Gene Census v91 [10] and DigSEE [36]. For each benchmark PPI network, labeled non‐driver genes were selected from network nodes after excluding the positive genes, NCG candidate cancer genes, genes recorded in OMIM disease annotations, genes listed in COSMIC CGC or the COSMIC coding mutation catalogue, and genes annotated to KEGG Pathways in Cancer. Only genes passing all exclusion steps and having at least one PPI interaction were retained as stringent negative samples, while the remaining non‐positive genes were treated as unlabeled.

Pan‐cancer multi‐omics profiles were derived from TCGA across 16 cancer types. For each gene, we used MF, CNA, differential DNA methylation, and differential gene expression. Each omics type was represented across the 16 cancer types, yielding a 64‐dimensional feature vector per gene.

PPI networks were obtained from CPDB [37], STRINGdb [38], MULTINET [39], PCNet [40], IRefIndex [41], and IRefIndex2015 [42]. Gene identifiers were standardized to official gene symbols before network construction. Interactions with confidence scores greater than 0.5 and 0.85 were retained for CPDB and STRINGdb, respectively. For IRefIndex, only binary interactions between human proteins were included. MULTINET and IRefIndex2015 were obtained from the HotNet2 resource, whereas PCNet followed the EMOGI construction protocol. After alignment of labels, features, and networks, dataset statistics were summarized in Table 1. Detailed formulations about feature similarity and CRISPR analysis are provided in the Supplementary Sections S15 and S16.

### 4.2 The DRIVE Model

We formulate cancer driver gene identification as a semi‐supervised node classification task on the omics‐level gene graph *G*  =  (*V*, *E*, **F**), where *V* denotes the set of *N* genes and *E* represents protein‐protein interactions (PPI) between gene pairs. The multi‐omics input is represented by a feature tensor $F\in R^{N\times M\times C}$, where *M* is the number of omics types and *C* is the number of cancer types. For gene *v_i_*, its feature vector is denoted as $f_{i}^{m}\in R^{C}$, where m∈M={mf,cna,meth,ge}, and |M|=M The node set comprises labeled genes $V_{L}=V_{L}^{+}\cup \;V_{L}^{-}$ and unlabeled genes *V_U_* =  *V*∖*V_L_*, where $V_{L}^{+}$ and $V_{L}^{-}$ denote known driver and non‐driver genes, respectively. Given *G*, DRIVE learns a parameterized model *f*_θ_(·) that integrates multi‐omics features and PPI topology to infer the driver probability ${\hat{y}}_{i}\in \left[0,1\right]$ for each gene, and ranks unlabeled genes according to this probability.

### 4.3 Module 1: Dual‐View Disentangled Graph Attention Network

The first module learns an omics‐level representation for each gene while reducing signal mixing caused by heterophilic PPI topology. For gene *v_i_*, each omics profile is first projected by an independent nonlinear encoder into a latent single‐omics node embedding $x_{i}^{m}\in R^{d}$. The encoder parameterization is detailed in Section S17.1.

#### 4.3.1 Heterophily Disentanglement Layer

PPI networks contain extensive interactions between genes with distinct driver labels and omics alteration patterns [4, 12, 60]. Direct aggregation over the original neighborhood may therefore mix biologically discordant signals and compromise the discriminability of gene representations. To address this issue, DRIVE decomposes the original PPI topology into two complementary relation views according to the omics consistency of connected genes. For each PPI connecting genes $(v_{i},v_{j})\in E$, the embedding consistency score $s_{ij}^{m}$ is defined as:

(1) $$s_{ij}^{m}=\mathrm{cosine}\;\left(x_{i}^{m},x_{j}^{m}\right)=\frac{x_{i}^{m}\cdot x_{j}^{m}}{\left|\left|x_{i}^{m}\right|\right|\left|\left|x_{j}^{m}\right|\right|}\;$$

where a larger $s_{ij}^{m}$ indicates stronger concordance between the omics alteration profiles of genes *v_i_* and *v_j_* under omics type *m*. Given a threshold τ, each PPI edge is assigned to one of the two views according to:

(2) $$\left(v_{i},v_{j}\right)\in \left\{\begin{array}{cc} E_{m}^{tight}, & s_{ij}^{m}\geq \tau ,\\ E_{m}^{loose}, & s_{ij}^{m}<\tau . \end{array}\right.$$

The two edge subsets define the tight and loose omics‐level graph views. Here, τ is a fixed hyperparameter selected as 0.5 based on validation performance. Because the latent embeddings are updated during optimization, DRIVE reconstructs the tight and loose views once per epoch using the current embeddings. The original PPI edge set remains fixed, while individual edges can switch between the two views. During inference, model parameters are fixed, and the final views are constructed once from the learned embeddings before aggregation and prediction. The tight view emphasizes interactions between genes with concordant molecular profiles, whereas the loose view preserves lower‐consistency interactions that can still provide complementary context in heterophilic biological networks. The full graph‐view construction is provided in Section S17.2.

#### 4.3.2 View‐Oriented Attention Layer

DRIVE next performs symmetric multi‐head attention within each disentangled graph view. For every omics branch and attention head, query, key, and value projections are used to construct a symmetric pairwise interaction encoding for connected genes. The resulting view‐specific attention coefficients are normalized over the corresponding neighborhood. For gene *v_i_*, attention head *h* aggregates neighbors in omics type *m* and graph view r∈{tight,loose} as:

(3) $$h_{i}^{m,r,h}=\sum_{v_{j}\in N_{i}^{m,r}}a_{ij}^{m,r,h}\;\cdot v_{j}^{m,h}$$

The query, key, and value projections, symmetric interaction encoding and attention normalization are formally defined in Section S17.3. Within each attention head, the tight‐view and loose‐view representations are combined according to their relative contribution, and the outputs of all heads are concatenated to form the graph‐derived representation. If a view‐specific neighborhood is empty, its aggregation is defined as a zero vector, while the other view continues to provide graph context. In parallel, a non‐graph branch directly transforms the original omics profile to preserve intrinsic molecular alterations. When both graph‐view neighborhoods are empty, the graph‐derived representation is zero and the gene representation is retained through the non‐graph branch. The multi‐head integration, non‐graph mapping and graph/non‐graph fusion operations are detailed in Section S17.3. Finally, DRIVE integrates the graph‐derived and non‐graph representations to obtain the omics‐level gene representation $h_{i}^{m}$.

### 4.4 Module 2: Contrastive Multi‐Omics Fusion

The four omics‐level representations ${\left\{h_{i}^{m}\right\}}_{m\in M}$ can contain molecular signals shared across omics types together with evidence retained within individual omics. DRIVE decomposes these heterogeneous representations into an omics‐common component and four omics‐specific components, and then combines them through gene‐adaptive integration.

#### 4.4.1 Learning Omics‐Common Gene Representation

A shared attention network assigns a normalized, gene‐dependent weight $\gamma_{i}^{m}$ to each omics‐level representation, allowing the relative contributions of MF, CNA, METH, and GE to vary across genes. The omics‐common representation is computed as

(4) $$z_{i,c}=\sum_{m\in M}\gamma_{i}^{m}\cdot h_{i}^{m}\;$$

The attention scoring and normalization functions used to derive the omics weights are provided in Section S17.4. To strengthen cross‐omics consistency, DRIVE further aligns each omics‐level representation with the omics‐common representation of the same gene using a contrastive learning objective [61]. Representations from the same gene construct positive pairs $\left(h_{i}^{m},z_{i,c}\right)$, whereas common representations from different genes construct negative pairs ${\left(h_{i}^{m},z_{j,c}\right)}_{j\neq i}$. These negative pairs are defined by gene identity and do not use non‐driver annotations, allowing both labeled and unlabeled genes to contribute to representation learning. Using an InfoNCE [62] objective, the contrastive loss is defined as:

(5) $$\begin{array}{c} L_{i}^{cl}=-\frac{1}{\left|M\right|}\sum_{m\in M}\log\frac{\exp\left[\pi \left(h_{i}^{m},z_{i,c}\right)/t\right]}{\begin{array}{c} \exp\left[\pi \left(h_{i}^{m},z_{i,c}\right)/t\right]\\ +\sum_{j\in V_{i}^{-}}\exp\left[\pi \left(h_{i}^{m},z_{j,c}\right)/t\right] \end{array}} \end{array}$$

Here, π(·, ·) denotes cosine similarity, *t* is the temperature parameter, and $V_{i}^{-}$ is the sampled negative set for gene *v_i_*. Specifically, $V_{i}^{-}$ is constructed by randomly sampling genes from *V*∖{*v_i_*}, such that any gene other than *v_i_* can serve as a contrastive negative. Minimizing the contrastive loss aligns each omics‐level representation with the omics‐common representation of the same gene while separating representations of different genes [63], thereby preserving gene‐level discriminability and discouraging trivial global representation collapse.

#### 4.4.2 Learning Omics‐Specific Gene Representations

The common representation captures convergent molecular evidence, but source‐specific signals can also contribute to driver identification. DRIVE therefore derives an omics‐specific residual representation for each branch:

(6) $$z_{i,s}^{m}=h_{i}^{m}\;-z_{i,c}$$

The residual retains the component of an individual omics type after removal of the common signal. To reduce redundancy among the four residual spaces, DRIVE applies a soft orthogonality constraint:

(7)

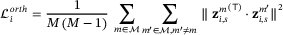




Minimizing the orthogonality loss explicitly penalizes pairwise inner products among the omics‐specific residual representations [64], encouraging them to occupy complementary latent directions and thereby reducing cross‐omics redundancy and discouraging directional collapse of the source‐specific components.

#### 4.4.3 Adaptive Representation Integration Layer

After obtaining the omics‐common representation and the four omics‐specific representations, DRIVE estimates their relative contributions with a shared scoring function. The resulting normalized coefficients are gene‐specific and sum to one. The final fused representation is:

(8) $$z_{i}=\lambda_{i,c}\;\cdot z_{i,c}+\sum_{m\in M}\lambda_{i,s}^{m}\cdot z_{i,s}^{m}$$

Here, λ_*i*,*c*_ denotes the contribution of the omics‐common representation and $\lambda_{i,s}^{m}$ the contribution of each omics‐specific representation. The scoring and coefficient‐normalization procedures are detailed in Section S17.5. This adaptive integration allows DRIVE to assign different combinations of molecular evidence to individual genes. The fused representation **z**_*i*_ is subsequently passed to the prediction module.

### 4.5 Module 3: Prediction and Joint Optimization

The final fused representation is mapped by a two‐layer prediction head with sigmoid activation to obtain the driver probability ${\hat{y}}_{i}$. The complete prediction function is provided in Section S17.6. Training combines supervised objectives on labeled genes with self‐supervised representation objectives defined over the entire graph (including unlabeled genes).

#### 4.5.1 Supervised Prediction Objectives

Because known drivers constitute a minority of labeled genes, DRIVE applies focal loss [65] to increase the contribution of difficult and minority‐class samples. Candidate discovery also depends on reliable gene ranking. For the pairwise margin loss, positive genes are labeled drivers from the current training fold, whereas negative genes are labeled non‐driver genes from the same training fold. During training, 50% of the available labeled non‐driver genes are randomly sampled for pair construction, and validation, test, and unlabeled genes are excluded. A pairwise margin‐ranking loss [66] therefore encourages each labeled driver to receive a higher probability than labeled non‐driver genes by a predefined margin. The supervised objective combines these focal and ranking components. Their complete definitions are provided in Section S17.7.

#### 4.5.2 Self‐Supervised Representation Objectives

The contrastive and orthogonality losses provide representation‐level supervision for all genes, including unlabeled nodes. The contrastive objective aligns each omics‐level representation with the omics‐common representation of the same gene, whereas the orthogonality objective reduces redundancy among omics‐specific residual components. The complete self‐supervised objective is defined in Section S17.7.

#### 4.5.3 Joint Optimization

DRIVE jointly optimizes the supervised prediction objectives and the self‐supervised representation objectives:

(9) $$L=L^{SL}+\eta \times L^{SSL}$$

Here, η controls the contribution of self‐supervised representation learning. Through joint optimization, focal and margin‐ranking losses guide discriminative driver probability estimation on labeled genes, while contrastive alignment and orthogonality regularization shape the multi‐omics representation space using both labeled and unlabeled nodes. After training, the predicted driver probability is used as the DRIVE driver score, and unlabeled genes are ranked in descending order to prioritize previously unrecognized cancer driver candidates.

### 4.6 Comparison Methods

We compared DRIVE with ten representative baselines: GCN [25], EMOGI [27], SMG [35], SGCD [30], deepCDG [31], MODCAN [33], GRAFT [28], MFC‐GCN [43], TREE [29], and MNDGNN [44]. These methods cover conventional graph neural networks, multi‐omics integration, heterophily‐aware graph modeling and graph representation learning. For controlled benchmark comparison, all methods used the same 64 dimensional MF, CNA, METH and GE features, identical gene labels and data splits, and one benchmark PPI network per run. For methods supporting additional biological networks, these additional network inputs were not used in this comparison. For every baseline, the optimal hyperparameter configuration was determined by grid search on the corresponding validation folds, and the selected configuration was fixed before test evaluation.

### 4.7 Performance Evaluation

Prediction performance was evaluated using the area under the precision‐recall curve (AUPRC), the area under the receiver operating characteristic curve (AUROC), F1 score and accuracy (ACC). All methods were evaluated under the same semi‐supervised fivefold cross‐validation protocol on the labeled gene set. In each fold, labels for the held‐out genes were masked during model training and used only for final evaluation, whereas graph topology and multi‐omics features for all genes were retained. Hyperparameters were selected using validation data and fixed during test evaluation. The view threshold τ, contrastive temperature *t*, and self‐supervised coefficient η were treated as fixed hyperparameters and selected according to validation performance through systematic sensitivity analyses, yielding τ  =  0.5, *t*  =  0.5, and η  =  0.3. Gene level model interpretation was performed using GNNExplainer [48], with detailed mask optimization and attribution aggregation procedures provided in Supplementary Material.

## 5 Conclusion

We developed DRIVE, a disentangled graph representation framework for prioritizing cancer driver genes from heterogeneous multi‐omics network data. DRIVE addresses three coupled challenges, heterophilic PPI topology, modality‐specific molecular evidence and sparse gene annotations, by combining dual‐view topology disentanglement, contrastive multi‐omics fusion and joint supervised/self‐supervised optimization. Across six benchmark PPI networks, DRIVE achieved a mean AUPRC of 0.9204 and a mean AUROC of 0.9704, outperforming ten graph‐learning and multi‐omics baselines. Ablation analysis, CKA representation diagnostics, masked‐driver recovery and label‐ratio sensitivity experiments showed that each design element contributed to the performance gains. Biological validation further showed that DRIVE PCDGs were enriched near known drivers, had stronger DepMap CRISPR dependency support than candidates from comparison methods and were linked to immune‐regulatory pathways, NF‐κB and T‐cell signaling, checkpoint‐related processes and drug sensitivity. These results support DRIVE as a robust and biologically grounded framework for nominating candidate cancer driver genes and generating testable hypotheses for precision oncology.

## Author Contributions

X.G.: conceptualization, data curation, methodology, software, validation, investigation, formal analysis, visualization, writing – original draft, writing – review & editing. J.L.: data curation, formal analysis, writing – review & editing. M.S.: investigation, formal analysis, writing – review & editing. P.Y.: writing – review & editing. T.M.: writing – review & editing. R.Z.: writing – review & editing. C.Z.: writing – review & editing. M.Z.: conceptualization, methodology, supervision, resources, writing – review & editing. Y.Z.: conceptualization, supervision, funding acquisition, project administration, resources, writing – review & editing. All authors reviewed the manuscript.

## Conflicts of Interest

The authors declare no conflicts of interest.

## Supporting information




**Supporting File 1**: smll72608‐sup‐0001‐SuppMat.docx.

**Supporting File 2**: advs77970‐sup‐0002‐SuppMat.xlsx.

## Data Availability

The experimental datasets are publicly available via Zenodo at https://zenodo.org/records/21470286. The source code about DRIVE is publicly available in the GitHub repository at https://github.com/rijianxiaoshou/DRIVE.
