# Supplementary material for: Disentangling Heterogeneous Molecular Networks for Multi‐Omics‐Driven Cancer Driver Discovery
Source: Adv Sci (Weinh). 2026 Sep 27:e77970. Online ahead of print. doi: 10.1002/advs.77970 (PMC13616402; doi:10.1002/advs.77970)
Supplement: Supplementary file 1 [file ADVS-9999-e77970-s001.docx]

**Disentangling Heterogeneous Molecular Networks for Multi-Omics-Driven Cancer Driver Discovery (Supplementary Materials)**

Contents

[S1 Benchmark dataset overview	2](#_Toc238674197)

[S2 Hyperparameter sensitivity and optimization stability	4](#_Toc238674198)

[S3 Robustness to incomplete PPI topology	8](#_Toc238674199)

[S4 Negative sample definition sensitivity analysis	9](#_Toc238674200)

[S5 Baselines and experimental settings	10](#_Toc238674201)

[S6 Training-positive overlap analysis for external cancer driver gene sets	13](#_Toc238674202)

[S7 Generalization across biological network structures and cancer contexts	13](#_Toc238674203)

[S8 GNNExplainer based interpretation of DRIVE predictions	16](#_Toc238674204)

[S9 The unique candidates identified by DRIVE	19](#_Toc238674205)

[S10 Prior evidence stratification and comparative biological characterization of DRIVE candidates	20](#_Toc238674206)

[S11 Driver associated genomic evidence supporting DRIVE PCDGs	24](#_Toc238674207)

[S12 Immunotherapy-related material	26](#_Toc238674208)

[S13 Sensitivity analysis of representative-gene prioritization and random-gene-set controls	27](#_Toc238674209)

[S14 Covariate adjusted immune associations and clinical relevance of DRIVE PCDGs	32](#_Toc238674210)

[S15 Feature and CKA similarity equations	36](#_Toc238674211)

[S16 Network and CRISPR analysis of PCDGs	37](#_Toc238674212)

[S17 Detailed mathematical equation of DRIVE	39](#_Toc238674213)

[Reference	43](#_Toc238674214)

### S1 Benchmark dataset overview

In this study, we collected and curated publicly released clinical pan cancer data to construct six benchmark datasets for cancer driver gene identification. . Our data collection and preprocessing pipeline consist of three major parts: gene labels, multi-omics data, and protein-protein interaction networks. The remainder of this section introduces these parts in order.

We integrated high confidence evidence from the Network of Cancer Genes (NCG v6.0), COSMIC Cancer Gene Census (CGC v91), and DigSEE to collect driver genes that have been publicly reported in the literature and curated databases as positive samples. For each benchmark PPI network, labeled non-driver genes were selected from network nodes after excluding the positive genes, NCG candidate cancer genes, genes recorded in OMIM disease annotations, genes listed in COSMIC CGC or the COSMIC coding mutation catalogue, and genes annotated to KEGG Pathways in Cancer. Only genes passing all exclusion steps and having at least one PPI interaction were retained as stringent negative samples, while the remaining non-positive genes were treated as unlabeled.

The pan cancer multi-omics data were obtained from 29,446 samples across 16 cancer cohorts in The Cancer Genome Atlas (TCGA), including Bladder Urothelial Carcinoma (BLCA), Breast Invasive Carcinoma (BRCA), Cholangiocarcinoma (CHOL), Colon Adenocarcinoma (COAD), Esophageal Carcinoma (ESCA), Head and Neck Squamous Cell Carcinoma (HNSC), Kidney Renal Clear Cell Carcinoma (KIRC), Kidney Renal Papillary Cell Carcinoma (KIRP), Liver Hepatocellular Carcinoma (LIHC), Lung Adenocarcinoma (LUAD), Lung Squamous Cell Carcinoma (LUSC), Pancreatic Adenocarcinoma (PAAD), Prostate Adenocarcinoma (PRAD), Rectum Adenocarcinoma (READ), Thyroid Carcinoma (THCA), and Uterine Corpus Endometrial Carcinoma (UCEC). For each gene, we constructed four types of molecular features, including mutation frequency, copy number aberration, differential DNA methylation, and differential gene expression. The details are as follows.

(1) Mutation frequency (MF). The functional mutation burden of each gene across cancer types was extracted from single nucleotide variant (SNV) records. We counted non silent mutations for each gene and normalized them by exon length obtained from GENCODE to reduce gene length bias. The MF of gene $i$ in cancer type $c$ is defined as:

$$\begin{aligned} mf_{i}^{\left( c \right)}=\frac{1}{\left| P_{c} \right|}\sum_{p\in P_{c}} f_{p,i}\#(1) \end{aligned}$$

where $P_{c}$ denotes the patient cohort of cancer type $c$, $|P_{c}|$ is the number of samples in this cohort, and $f_{p,i}$ denotes the length normalized non silent mutation frequency of gene $i$ in patient $p$. Thus, the feature vector $\mathbf{f}_{i}^{mf}=\left\{ mf_{i}^{\left( c \right)} \right\}_{1}^{C},\mathbf{f}_{i}^{mf}\in\mathbb{R}^{C}$, characterizes the mutational activity of gene $i$.

(2) Copy number aberration (CNA). We considered both amplification and deletion events to quantify the cumulative CNA burden of each gene in each cancer type. The average CNA of gene $i$ in cancer type $c$ is defined as:

$$\begin{aligned} \mathrm{cna}_{i}^{\left( c \right)}=\frac{1}{\left| P_{c} \right|}\sum_{p\in P_{c}} \mathbb{I}\left( a_{p,i}\neq0 \right)\#\left( 2 \right) \end{aligned}$$

where $a_{p,i}$ denotes the CNA status of gene $i$ in patient $p$, and a nonzero value indicates amplification or deletion. $\mathbb{I(\cdot)}$ is the indicator function, which equals 1 if the condition is satisfied and 0 otherwise. The feature vector $\mathbf{f}_{i}^{cna}=\left\{ \mathrm{cna}_{i}^{\left( c \right)} \right\}_{1}^{C},\mathbf{f}_{i}^{cna}\in\mathbb{R}^{C}$, represents the structural perturbation level of gene $i$.

(3) Differential DNA methylation (METH). We used Illumina HumanMethylation450K array signals to measure promoter methylation shifts in tumor tissues relative to adjacent normal tissues, thereby reducing the effects of tissue background and individual variability. The METH feature of gene $i$ in cancer type $c$ is defined as:

$$\begin{aligned} \mathrm{meth}_{i}^{\left( c \right)}=\frac{1}{\left| P_{c} \right|}\sum_{p\in P_{c}} \left( \beta_{p,i}^{+}-\beta_{p,i}^{-} \right)\#\left( 3 \right) \end{aligned}$$

where $\beta_{p,i}^{+}$ and $\beta_{p,i}^{-}$ denote the promoter methylation levels of gene $i$ in tumor and adjacent normal tissues from patient $p$, respectively. Therefore, the feature vector $\mathbf{f}_{i}^{meth}=\left\{ \mathrm{meth}_{i}^{\left( c \right)} \right\}_{1}^{C},\mathbf{f}_{i}^{meth}\in\mathbb{R}^{C}$, captures the epigenetic alteration level of gene $i$.

(4) Differential gene expression (GE). We used RNA-seq expression profiles to characterize the magnitude of expression reprogramming in tumors relative to normal tissues. Genes with zero values in more than 10% of samples were filtered out to reduce noise, and log2 fold-change was used to stabilize the absolute expression difference. The GE feature of gene $i$ in cancer type $c$ is defined as:

$$\begin{aligned} \mathrm{ge}_{i}^{\left( c \right)}=\frac{1}{\left| P_{c} \right|}\sum_{p\in P_{c}} \log_{2} \left( \frac{E_{p,i}^{+}+\epsilon}{E_{p,i}^{-}+\epsilon} \right)\#\left( 4 \right) \end{aligned}$$

where $E_{p,i}^{+}$ and $E_{p,i}^{-}$ denote the expression abundance of gene $i$ in tumor and paired normal samples from patient $p$, respectively, and $\epsilon$ is a small constant introduced to avoid zero division. The feature vector $\mathbf{f}_{i}^{ge}=\left\{ \mathrm{ge}_{i}^{\left( c \right)} \right\}_{1}^{C},\mathbf{f}_{i}^{ge}\in\mathbb{R}^{C}$ represents the log fold change of gene expression.

Protein-protein interaction (PPI) networks were collected from CPDB, STRINGdb, MULTINET, PCNet, IRefIndex, and IRefIndex2015. Specifically, CPDB was obtained from ConsensusPathDB, STRINGdb from the STRING database, MULTINET and IRefIndex2015 from the public HotNet2 repository, IRefIndex from the IRefIndex database, and PCNet followed the version used in EMOGI^1^. To remove ambiguity in gene identifiers across databases, we unified gene symbols through Ensembl ID based mapping. We then filtered interactions according to the evidence strength of each database. For CPDB, only interactions with confidence scores above 0.5 were retained, while the threshold for STRINGdb was set to 0.85. For IRefIndex, interactions were restricted to binary interactions between human proteins. MULTINET, IRefIndex 2015, and PCNet were kept consistent with their original construction protocols.

To quantify the degree of heterophily in the benchmark PPI networks, we calculated the k-hop node homophily ratio for each dataset. This ratio is calculated as the proportion of k-hop neighboring nodes belonging to the same class. Let $V$ denote the set of genes and $\mathcal{N}_{i}^{k}$ denote the k-hop neighborhood of gene $v_{i}$. The k-hop node homophily ratio was calculated as:

$$\begin{aligned} h^{k}=\frac{1}{\left| V \right|}\sum_{v_{i}\in V} \frac{\left| \left\{ v_{j} | v_{j}\in\mathcal{N}_{i}^{k},y_{j}=y_{i} \right\} \right|}{\left| \mathcal{N}_{i}^{k} \right|}\#(5) \end{aligned}$$

where $y_{i}$ denotes the label of gene $v_{i}$. A lower value of $h^{k}$, approaching 0, indicates stronger heterophilic topology, suggesting that neighboring genes tend to have different labels. In contrast, a higher value of $h^{k}$, approaching 1, indicates stronger homophilic topology, suggesting that neighboring genes tend to share the same label. The complete k-hop homophily results from 1-hop to 6-hop neighborhoods are reported in **Table S3**. These results show that the benchmark PPI networks provide limited label-consistent neighborhoods for conventional graph aggregation. Under such heterophilic topology, messages propagated from neighboring genes may mix driver and non-driver signals within the same representation space, leading to cross class feature entanglement and reduced discriminability.

Next, we systematically examined the heterogeneity of multi omics evidence by evaluating all possible combinations of multi-omics data. For each gene $v_{i}$, the four omics types, including mutation frequency, DNA methylation, gene expression and copy number aberration, were each represented as a 16-dimensional vector $\mathbf{f}_{i}^{m}\in\mathbb{R}^{16}$. Let $\mathcal{M=\{}mf,cna,meth,ge\}$ denote the full omics set, $m\mathcal{\in M}$. For any non-empty subset $\mathcal{S}\subseteq\mathcal{M}$, the corresponding multi-omics feature of gene $v_{i}$ was constructed by concatenating the selected omics vectors, $\mathbf{f}_{i}^{\mathcal{S}}=\parallel_{m\in\mathcal{S}}\mathbf{f}_{i}^{m}\in\mathbb{R}^{16|\mathcal{S}|}$. This procedure produced $2^{4}-1=15$ omics combinations, ranging from 16-dimensional single-omics inputs, 32-dimensional two-omics inputs, 48-dimensional three-omics inputs to 64-dimensional four-omics inputs.

For each benchmark PPI network, we constructed an attributed graph $G^{\mathcal{S}}=(V,E,\mathbf{F}^{\mathcal{S}})$, where $\mathbf{F}^{\mathcal{S}}\boldsymbol{\in}\mathbb{R}^{N\times16|\mathcal{S}|}$. A two-layer GCN was then trained on each graph to quantify the predictive contribution of each omics combination. Complete AUPRC and AUROC results are reported in **Table S9**. These results indicate that different omics layers contain partially redundant or conflicting evidence. Simple concatenation of omics features does not consistently yield performance gains and can even impair predictive performance, highlighting the need for adaptive multi omics fusion.

### S2 Hyperparameter sensitivity and optimization stability

#### S2.1 Hyperparameter sensitivity analysis

To evaluate the robustness of DRIVE to its principal hyperparameters, we systematically varied the parameters controlling dynamic graph construction, contrastive learning, and joint optimization while keeping the remaining settings unchanged. The view threshold τ in main text Eq. 2 was evaluated over {0.3,0.4,0.5,0.6,0.7}, the contrastive temperature t in Eq. 5 over {0.1,0.2,0.5,1.0,2.0}, and the self-supervised coefficient η in Eq. 9 over {0,0.1,0.3,0.5,0.7,1.0}. AUPRC and AUROC were evaluated separately on all six benchmark PPI networks.

As shown in Supplementary Fig. S1, DRIVE remained stable across intermediate settings of all three parameters. For the graph view threshold, excessively small values make the tight view less selective, whereas larger values assign more interactions to the loose view. The six networks mean reached its best overall performance at τ=0.5, which was therefore used throughout the main experiments. The threshold remains fixed during training; dynamic topology results from changes in the learned embeddings rather than changes in τ. For contrastive learning, t=0.5 provided the best overall balance between similarity discrimination and overly sharp or diffuse contrastive weighting. For joint optimization, performance improved substantially when the self-supervised objective was introduced from η=0, reached its maximum around η=0.3, and gradually decreased as the representation objective received excessive weight. We therefore fixed t=0.5 and η=0.3 for the final model.

**Supplementary Fig. S1 | Hyperparameter sensitivity of DRIVE across six PPI networks.** a,b, AUPRC and AUROC under different view thresholds τ in Eq. 2. c,d, AUPRC and AUROC under different contrastive temperatures t in Eq. 5. e,f, AUPRC and AUROC under different self-supervised coefficients η in Eq. 9. Thin curves represent individual PPI networks and the thick curve represents the six networks mean. Vertical dotted lines indicate the parameter values used in the final model, τ=0.5, t=0.5, and η=0.3.

#### S2.2 Optimization stability experiment

We next examined the optimization dynamics of DRIVE over 30 epochs. Supplementary Fig. S2 shows the training and validation losses for all six PPI networks. Both losses decreased substantially during early optimization and subsequently approached stable plateaus. Validation loss exhibited small network dependent fluctuations but showed no sustained divergence from the training trajectory. The validation selected epochs were 22, 27, 19, 26, 23, and 25 for CPDB, IRefIndex, IRefIndex2015, MULTINET, PCNet, and STRINGdb, respectively.

The corresponding validation AUPRC and AUROC trajectories are shown in Supplementary Fig. S3. Both metrics improved rapidly during early epochs and then gradually stabilized. The six networks followed distinct convergence trajectories, consistent with their different graph densities and interaction structures. The test values annotated in the figure are the fivefold test means and are shown only as reference values; model selection was based on validation performance.

**Supplementary Fig. S2 | Network specific optimization dynamics of DRIVE.** Training and validation loss trajectories over 30 epochs are shown separately for the six benchmark PPI networks. Vertical dotted lines indicate the epochs selected according to validation performance.

**Supplementary Fig. S3 | Predictive convergence of DRIVE across six PPI networks.** Validation AUPRC and AUROC trajectories over 30 epochs are shown for each benchmark network. Vertical dotted lines indicate the validation selected epochs. Annotated AUPRC and AUROC values denote the corresponding fivefold test means and are provided as reference values rather than validation trajectories.

Finally, we quantified the stability of dynamic graph construction using the edge reassignment rate between consecutive epochs. For omics branch $m$, let $a_{ij,m}^{\left( e \right)}\in\{tight,loose\}$ denote the view assignment of PPI edge $\left( i,j \right)\in E$ at epoch $e$. The edge reassignment rate at epoch $e$ was defined as:

$$\begin{aligned} R^{\left( e \right)}=\frac{100}{M\left| E \right|}\sum_{m=1}^{M} \sum_{\left( i,j \right)\in E} \mathbb{I}\left( a_{ij,m}^{\left( e \right)}\neq a_{ij,m}^{\left( e-1 \right)} \right)\#\left( 6 \right) \end{aligned}$$

where $M=4$ denotes the number of omics branches, $|E|$ is the number of original PPI interactions, and $\mathbb{I}(\cdot)$ is the indicator function. Thus, $R^{\left( e \right)}$ represents the percentage of original PPI edges whose tight or loose assignments changed relative to the preceding epoch, averaged across the four omics branches. As shown in Supplementary Fig. S4, reassignment rates were relatively high during early training and progressively decreased as optimization proceeded. The horizontal 2% line is included as a visual reference rather than a formal convergence criterion. By the validation selected epochs, edge reassignment had reached low levels across all networks, indicating that DRIVE actively reorganizes PPI relations during early representation learning and progressively converges toward stable graph partitions.

**Supplementary Fig. S4 | Stabilization of dynamically constructed graph views across six PPI networks.** Edge reassignment rate quantifies the proportion of original PPI interactions whose tight or loose assignments changed relative to the preceding epoch. Vertical dotted lines indicate the validation selected epochs. The horizontal dotted line at 2% is shown as a visual reference. The progressive decrease in reassignment rate indicates stabilization of the dynamic graph partitions during optimization.

### S3 Robustness to incomplete PPI topology

To assess sensitivity to incomplete network structure, we randomly removed 5%, 10%, or 20% of the original PPI interactions from each benchmark network before training, while keeping the node set, multi-omics features, gene labels, data splits, and hyperparameters unchanged. DRIVE was retrained on each perturbed network, with dynamic tight and loose views reconstructed from the remaining PPI edges. As shown in Supplementary Fig. S5, performance decreased gradually with increasing edge removal but remained stable under moderate structural perturbation. At 20% edge removal, mean AUPRC decreased from 0.9204 to 0.9028 and mean AUROC from 0.9704 to 0.9626.

**Supplementary Fig. S5 | Robustness of DRIVE to incomplete PPI networks.** a, AUPRC of DRIVE after randomly removing 0%, 5%, 10%, or 20% of the original PPI interactions from each benchmark network. b, Corresponding AUROC results. Each curve denotes one PPI network, and the mean curve summarizes performance across all six networks.

### S4 Negative sample definition sensitivity analysis

The original DRIVE benchmark uses a conservative exclusion based on strategy for labeled non driver construction. Positive genes were collected from NCG v6.0, COSMIC CGC v91, and DigSEE. For each benchmark PPI network, labeled non-driver genes were selected from network nodes after excluding the positive genes, NCG candidate cancer genes, genes recorded in OMIM disease annotations, genes listed in COSMIC CGC or the COSMIC coding mutation catalogue, and genes annotated to KEGG Pathways in Cancer. Only genes passing all exclusion steps and having at least one PPI interaction were retained as stringent negative samples, while the remaining non-positive genes were treated as unlabeled.

To evaluate the sensitivity of DRIVE to residual uncertainty in negative labels, we constructed three experimental settings. Original strategy S1 corresponds to the negative sample definition used throughout the main experiments. Stringent strategy S2 applies an additional CancerMine based screening step to the S1 labeled negative set. CancerMine provides literature mined evidence for cancer drivers, oncogenes, and tumor suppressors. Genes receiving such evidence were removed from the labeled negative pool and retained as unlabeled genes rather than being reassigned as positive samples. 50% negative subset strategy S3 randomly retains only 50% of the available S1 labeled non driver genes for supervised training, reducing the amount of negative supervision while leaving the positive label definition unchanged. DRIVE was retrained independently under each setting across all six benchmark PPI networks using the same multi omics features, cross validation protocol, and model configuration. AUPRC and AUROC were used for evaluation. Relative performance change for an alternative setting $S_{k}$ was calculated against S1 as

$$\begin{aligned} \Delta_{\mathrm{rel}}\left( S_{k} \right)=\frac{M\left( S_{k} \right)-M\left( S_{1} \right)}{M\left( S_{1} \right)}\times100\%\#\left( 7 \right) \end{aligned}$$

where $M$ denotes AUPRC or AUROC. As shown in Supplementary Fig. S6, performance remained highly stable under both alternative negative sample settings. Mean AUPRC changed from 0.9204 in S1 to 0.9234 in S2 and 0.9171 in S3. The corresponding mean AUROC values were 0.9704, 0.9720, and 0.9689. Relative to S1, S2 increased mean AUPRC by 0.32% and mean AUROC by 0.17%, while S3 decreased them by only 0.36% and 0.15%, respectively.

**Supplementary Fig. S6 | Sensitivity of DRIVE to alternative negative sample definitions.** **a,** AUPRC across six PPI networks under Original strategy S1, Stringent strategy S2, and 50% negative subset strategy S3 settings. **b,** Corresponding AUROC results. Bars show absolute performance and lines show the relative change of S2 and S3 compared with S1. S2 further excludes labeled negative genes carrying CancerMine cancer related evidence, whereas S3 retains only 50% of the original labeled negative genes for supervised training. The horizontal dashed line represents zero relative change.

The consistent improvement under the stricter S2 setting suggests that additional exclusion of genes with literature based on cancer evidence can modestly reduce residual negative label noise. Conversely, the limited decrease under S3 shows that DRIVE does not strongly depend on the full labeled non driver set and remains stable when negative supervision is substantially reduced. Together, these analyses support the robustness of DRIVE to plausible uncertainty in negative sample definition and sampling density.

### S5 Baselines and experimental settings

DRIVE was compared with ten representative baseline methods covering conventional graph convolution, explainable graph learning, self-supervised graph representation learning, heterophily aware graph learning, multi omics fusion and transformer-based graph modeling.

- EMOGI^1^ is an explainable graph convolutional framework for cancer driver gene identification. Pan-cancer multi-omics profiles are coupled with PPI topology in a semi-supervised manner, allowing both molecular alterations and neighborhood structure to support prediction.
- GCN^2^ is a standard graph convolutional baseline for driver gene prediction. By propagating gene features over the PPI graph, local network context is incorporated into node representations for downstream classification.
- SMG^3^ is a self-supervised masked graph learning method for cancer gene identification. Pretraining is conducted by reconstructing masked nodes on multiomic PPI graphs, after which the learned encoder is adapted to the downstream prediction task.
- SGCD^4^ is a graph neural network tailored to heterophilic biological networks for driver gene identification. Representation separation and bimodal feature extraction are jointly employed, thereby reducing feature confusion while preserving topological and omics cues.
- deepCDG^5^ is a deep graph convolutional model for multi-omics integration in cancer driver gene identification. Different omics are encoded with shared parameters and then fused by attention, with residual graph propagation used to strengthen final prediction.
- MODCAN^6^ is a semi-supervised framework for tumor-specific driver gene identification. After subtype stratification, differential co-association graphs are constructed to reflect rewired gene relations, helping the model capture tumor heterogeneity more explicitly.
- GRAFT^7^ is a graph-aware fusion transformer for cancer driver gene prediction. Multiple biological networks are integrated with structural and functional signals, while edge-aware attention is used to model both nearby and long-range dependencies.
- MFC-GCN^8^ is a multi-network fusion model for driver gene identification. Cross-network representation learning is strengthened through contrastive objectives, and adaptive fusion is achieved with a mixture-of-experts mechanism.
- TREE^9^ is a Transformer based graph representation learning framework designed to integrate multi omics attributes with local and global structural encodings. It samples local graph structures and uses attention-based representation learning to capture long range gene dependencies.
- MNDGNN^10^ is a directed graph neural network originally developed for multiplex biological network integration. Its graph convolution incorporates neighbor diversity and degree diversity, together with sample augmentation for sparse labels. The original framework integrates PPI, protein complex, pathway, regulatory and kinase substrate networks.

All methods were evaluated under a semi-supervised setting using five-fold cross-validation on the labeled gene set. The dimensional of embedding was set to $d=64$, and the hidden dimensions of the attention scoring network and prediction head were set to $d_{a}=32$ and $d_{p}=32$, respectively. The number of graph propagation layers in DRIVE was set to 2. In the heterophily disentanglement layer, the threshold $\tau=0.5$. In the View Oriented Attention Layer, the number of attention heads $H=4$. During training, the coefficient $\eta$ of the loss function was set to 0.3. For focal loss, the class weight $\delta=0.85$ , and the focusing parameter$\mu=2$. For pairwise margin loss, the margin $\xi=0.1$. The loss weight $w_{1}=0.3$ and $w_{2}=0.5$. The balance coefficients $\alpha$ and $\beta$ were selected according to validation performance through sensitivity analyses and were fixed during test evaluation. We used the Adam optimizer with an initial learning rate of 0.0149 and weight decay of 5×10^−5^. The maximum number of training epochs was set to 30. The model was implemented with PyTorch and PyTorch Geometric, and all experiments were conducted on an NVIDIA GeForce RTX 5060 Ti GPU with 16 GB memory.

Prediction performance was quantified using AUPRC, AUROC, F1 score and accuracy (ACC). Let TP, FP, TN and FN denote true positives, false positives, true negatives and false negatives, respectively. For AUPRC, precision and recall were defined as:

$$\begin{aligned} precision=\frac{\mathrm{TP}}{TP+FP} \end{aligned}\left( 8 \right)$$

$$\begin{aligned} recall=\frac{\mathrm{TP}}{TP+FN} \end{aligned}(9)$$

$$\begin{aligned} AUPRC=\int precision d\left( \mathrm{recall} \right) \end{aligned}(10)$$

For AUROC, the true positive rate (TPR) and false positive rate (FPR) were defined as:

$$\begin{aligned} TPR=\frac{\mathrm{TP}}{TP+FN} \end{aligned}(11)$$

$$\begin{aligned} FPR=\frac{\mathrm{FP}}{FP+TN} \end{aligned}(12)$$

$$\begin{aligned} AUROC=\int TPR d\left( \mathrm{FPR} \right) \end{aligned}(13)$$

F1 score and ACC were defined as:

$$\begin{aligned} F1=\frac{2 \times precision \times recall}{precision + recall} \end{aligned}(14)$$

$$\begin{aligned} ACC=\frac{TP+TN}{TP+TN+FP+FN} \end{aligned}(15)$$

To provide complete numerical results, we report AUPRC, AUROC, F1 score and ACC for DRIVE and all baseline methods across the six benchmark PPI networks. All values are presented as mean ± standard deviation across the cross-validation runs (Table S4).

To ensure comparability, the primary benchmark standardized the observable inputs across all methods. Each method received the same gene set, the same 64-dimensional node features comprising MF, CNA, METH and GE across 16 cancer types, and the same single PPI network for a given benchmark dataset. The same labeled genes and fivefold cross validation splits were used for all methods, and performance was evaluated using identical AUPRC, AUROC, F1 and ACC definitions. Additional biological networks supported by multi network methods were excluded from this benchmark comparison so that performance differences primarily reflected modeling strategies rather than differences in input information. Multi network generalization is evaluated separately. Method specific architectural settings were retained according to the corresponding implementations. Hyperparameters were selected using only the validation portion of each training fold, following the recommended values or search ranges of the original implementations. The selected configuration was then fixed for test evaluation.

### S6 Training-positive overlap analysis for external cancer driver gene sets

DRIVE training positives were constructed from the known cancer gene subset of NCG v6.0, COSMIC Cancer Gene Census v91, and DigSEE. NCG v6.0 contains two explicitly separated categories, including 711 known cancer genes and 1,661 candidate cancer genes. Only the known cancer gene subset was incorporated into DRIVE training labels. Consequently, the direct overlap between NCG known cancer genes used for training and NCG candidate cancer genes used for external evaluation was zero.

Because genes in the external reference sets could still overlap with the other training resources, we further compared OncoKB, ONGene, NCG candidate cancer genes, and the Bailey et al. driver gene set with the complete training-positive union. For an external reference set $E$ and the complete training-positive set $P_{\text{train}}$, the raw overlap was defined as $O_{E}=E\cap P_{\text{train}}.$ The filtered external positive set was subsequently defined as $E_{\text{filtered}}=E\backslash P_{\text{train}}.$This filtering was applied before external AUPRC calculation. Therefore, $E_{\text{filtered}}\cap P_{\text{train}}=⌀$ for all four external reference sets.

As summarized in Supplementary Table S5, substantial overlap was observed before filtering for several curated cancer gene resources. Among 682 OncoKB genes, 362 overlapped the complete training-positive union, leaving 320 genes after filtering. Among 803 ONGene genes, 415 overlapped the training-positive union, leaving 388 genes. For the 1,661 NCG candidate cancer genes, 104 genes overlapped the complete training-positive union through training sources outside the NCG known subset, leaving 1,557 candidate genes. Among the 299 genes in the Bailey et al. set, 216 overlapped the complete training-positive union, leaving 83 genes. No retained external positive gene overlapped the DRIVE training-positive set.

Removal of training-positive overlap had the largest proportional effect on the Bailey et al., OncoKB, and ONGene sets. Consequently, these external evaluations contain relatively sparse positive classes compared with the full gene space of the corresponding PPI networks, and the reduced number of true positives was associated with lower AUPR. The NCG candidate cancer gene set requires a distinct interpretation. Its 1,661 genes are explicitly classified by NCG as candidate cancer genes rather than known cancer genes. The candidate set therefore provides less stringently established cancer-gene evidence. Its substantially larger retained positive set is consistent with the higher external AUPRC observed for NCG candidate genes, while the candidate annotations not equal to confirmed driver labels.

### S7 Generalization across biological network structures and cancer contexts

To further evaluate the applicability of DRIVE beyond the six benchmark PPI networks, we conducted additional experiments on diverse biological interaction networks and cancer-specific datasets. To isolate the effect of network structure, all compared methods used the same node set, the same omics features, and the same evaluation protocol within each additional experiment.

#### S7.1 Single-network experiments across diverse biological networks

We adopted the biological network collection released with MNDGNN^10^, which provides matched gene graphs for five network types: protein complex, KEGG pathway, RegNetwork, DawnNet, and kinase-substrate. Because several source networks were originally directed, all directed edges were converted into undirected edges before model training and evaluation, so that DRIVE, TREE, and MNDGNN were compared under a unified graph setting. The same node features, label construction principle, and evaluation metrics were used across all methods.

As shown in Supplementary Fig. S**7**, DRIVE achieved the best AUPRC and AUROC on all six single-network settings. The advantage was observed on both dense and sparse network types, indicating that the dual-view disentangling strategy of DRIVE is applicable beyond the original PPI setting.

**Supplementary Fig. S7 | Generalization across single biological networks.** a, AUPRC comparison of DRIVE, TREE, and MNDGNN on six single biological networks. b, AUROC comparison on the same networks.

#### S7.2 Multi-network experiments

We next evaluated generalization in the multi-network setting using the same MNDGNN network collection. The original multi-network versions of MNDGNN and TREE were compared with several DRIVE-based network integration strategies. Specifically, we evaluated:

1. **DRIVE single PPI**, which used only the PPI network;
2. **DRIVE edge union**, which merged all network edges into one graph;
3. **DRIVE sum fusion**, which summed prediction scores from separately trained network-specific DRIVE models;
4. **DRIVE mean fusion**, which averaged the prediction scores from separately trained network-specific DRIVE models;
5. **DRIVE attention**, which used adaptive attention-based fusion across network-specific representations or predictions.

As shown in Supplementary Fig. S8, the attention-based DRIVE variant achieved the best overall multi-network performance, with AUPRC of 0.9317 and AUROC of 0.9658. It also outperformed edge union, score summation, and score averaging. These results suggest that simple heuristic fusion can retain redundant or mutually inconsistent signals across networks, whereas adaptive integration is more effective in exploiting complementary network information.

**Supplementary Fig. S8 |** **Generalization in the multi-network setting.** Comparison of MNDGNN, TREE, and different DRIVE-based multi-network integration strategies, including single PPI, edge union, sum fusion, mean fusion, and attention-based fusion.

#### S7.3 Cancer-specific experiments

We further investigated cancer-specific generalization on six tumor types: **BRCA, COAD, LUAD, LIHC, BLCA, and THCA**. For the cancer-specific experiments, positive labels were reconstructed separately for each tumor type rather than reusing the complete pan-cancer positive set. A gene was assigned a positive label only if it was annotated as a driver for the corresponding cancer type in at least one of NCG v6.0, COSMIC CGC v91, or DigSEE and remained present after alignment with the biological network used for that experiment. This procedure yielded 152 positive genes for BRCA, 126 for COAD, 117 for LUAD, 93 for LIHC, 81 for BLCA, and 49 for THCA. The decreasing positive-set sizes therefore reflect the cancer-specific availability of curated driver annotations rather than artificial subsampling. Negative labels were constructed using the same stringent exclusion principle as in the main pan-cancer benchmark: genes with documented cancer relevance in NCG, COSMIC, OMIM, or cancer-associated KEGG pathways were excluded from the negative class, and the remaining eligible genes were then restricted to the nodes present in the corresponding cancer-specific graph.

As shown in Supplementary Fig. S9, DRIVE consistently achieved the best AUPRC and AUROC across all six cancer-specific datasets. This result indicates that DRIVE generalizes well across varying cancer contexts, label distributions, and disease-specific molecular backgrounds.

**Supplementary Fig. S9 | Cancer-specific generalization experiments.** a, AUPRC comparison of DRIVE, TREE, and MNDGNN across BRCA, COAD, LUAD, LIHC, BLCA, and THCA. b, AUROC comparison across the same cancer-specific settings. In the x-axis labels, denotes the number of positive driver labels available for that cancer type.

### S8 GNNExplainer based interpretation of DRIVE predictions

#### S8.1 GNNExplainer formulation

To interpret individual DRIVE predictions, we applied GNNExplainer^11^ to the trained model. For each target gene $i$, all DRIVE parameters were fixed and the final tight and loose graph views constructed during inference were retained throughout explanation. This prevents explanation masks from altering the graph partition itself and isolates the molecular and structural evidence used by the trained prediction function.

Let $f_{\theta}$ denote the trained DRIVE model and $\hat{y}_{i}$ its predicted class for gene $i$. GNNExplainer learns continuous feature and edge masks that preserve the original prediction while selecting a compact subset of molecular features and graph interactions. For the four omics branches $m\in\{\text{MF},\text{CNA},\text{METH},\text{GE}\}$ and graph views $v\in\{\text{tight},\text{loose}\}$, the masked inputs are defined as

$$\begin{aligned} \tilde{A}_{m,v}^{\left( i \right)}=A_{m,v}\odot\sigma\left( M_{E,m,v}^{\left( i \right)} \right)\#\left( 16 \right) \end{aligned}$$

$$\begin{aligned} \tilde{X}^{\left( i \right)}=X\odot\sigma\left( M_{X}^{\left( i \right)} \right)\#\left( 17 \right) \end{aligned}$$

where $M_{E,m,v}^{\left( i \right)}$ is the edge mask, $M_{X}^{\left( i \right)}$ is the feature mask, $\sigma\left( \cdot\right)$ is the sigmoid function and $\odot$ denotes element wise multiplication. The explanation masks are optimized by minimizing

$$\begin{aligned} \mathcal{L}_{\text{exp}}^{\left( i \right)}=-logP_{\theta}\left( \hat{y}_{i}\mid{\tilde{\mathcal{G}}}^{\left( i \right)},\tilde{X}^{\left( i \right)} \right)+\lambda_{E}\mathcal{R}_{E}+\lambda_{X}\mathcal{R}_{X}\#\left( 18 \right) \end{aligned}$$

where the first term preserves the original DRIVE prediction and $\mathcal{R}_{E}$ and $\mathcal{R}_{X}$ denote sparsity and entropy regularization applied to the edge and feature masks, respectively. This follows the standard GNNExplainer principle of identifying a compact explanatory subgraph and a restricted feature subset that retain the target prediction.

#### S8.2 Omics attribution

DRIVE contains 64 input molecular features, with 16 dimensions corresponding to each omics source. For target gene $i$, the normalized attribution of omics source $m$ was calculated by aggregating the GNNExplainer feature mask over its corresponding feature dimensions:

$$\begin{aligned} A_{i,m}=\frac{\sum_{k\in\mathcal{F}_{m}} \sigma\left( M_{X,k}^{\left( i \right)} \right)}{\sum_{m'} \sum_{k\in\mathcal{F}_{m'}} \sigma\left( M_{X,k}^{\left( i \right)} \right)}\#\left( 19 \right) \end{aligned}$$

where $\mathcal{F}_{m}$ denotes the 16 feature dimensions belonging to omics source $m$. Therefore, $\sum_{m} A_{i,m}=1$. Global omics contribution was obtained by averaging normalized gene specific attribution across the complete PCDG set $\mathcal{C}$:

$$\begin{aligned} \bar{A}_{m}=\frac{1}{\left| \mathcal{C} \right|}\sum_{i\mathcal{\in C}} A_{i,m}\#\left( 20 \right) \end{aligned}$$

#### S8.3 Network and graph view attribution

Edge importance was obtained from the optimized GNNExplainer edge masks. For visualization of representative genes, the six directly connected PPI neighbors with the highest normalized edge attribution were retained. The displayed edge scores therefore represent GNNExplainer attribution and do not correspond to original PPI confidence scores, embedding similarity, or VOA attention coefficients. To quantify the relative structural contribution of the two DRIVE graph views, edge attribution was grouped according to the fixed inference view assignment. For target gene $i$, the normalized contribution of view $v$ was calculated as

$$\begin{aligned} S_{i,v}=\frac{\sum_{m} \sum_{e\in E_{m,v}} \sigma\left( M_{E,m,v,e}^{\left( i \right)} \right)}{\sum_{v^{'}} \sum_{m} \sum_{e\in E_{m,v^{'}}} \sigma\left( M_{E,m,v^{'},e}^{\left( i \right)} \right)}\#\left( 21 \right) \end{aligned}$$

Global structural attribution was then calculated as

$$\begin{aligned} \bar{S}_{v}=\frac{1}{\left| C \right|}\sum_{i\in C} S_{i,v}\#\left( 22 \right) \end{aligned}$$

In our setting, $\left| C \right|=186$. The tight and loose values are therefore normalized within graph derived attribution and quantify the relative structural evidence carried by the two learned graph views.

#### S8.4 Result analysis

Supplementary Fig. S9 summarizes the GNNExplainer analysis. RELA and FYN were selected as representative high confidence candidate genes for local interpretation. For RELA, the strongest attributed PPI interaction was NFKB1 (0.94), followed by IKBKG (0.88), NFKBIA (0.82) and CHUK (0.78), forming an NF κB associated local neighborhood. Its molecular attribution was dominated by GE (0.40), followed by MF (0.25), CNA (0.18) and METH (0.17). FYN showed a distinct explanatory pattern, with GRB2 (0.92), SHC1 (0.79), SOS1 (0.74) and PIK3CG (0.69) receiving high edge attribution, while MF (0.36) and GE (0.29) provided the strongest molecular contributions.

Gene specific explanations further demonstrated that DRIVE does not rely on a single molecular source for all candidates. Among the representative examples, RELA was GE dominant, FYN was MF dominant, ANLN was CNA dominant and NR3C1 was METH dominant. At the global level, normalized omics contributions were 0.334 for MF, 0.284 for CNA, 0.200 for METH and 0.183 for GE. Thus, mutation and copy number information provided the strongest average attribution, while all four molecular sources contributed to the candidate set.

Graph view attribution showed a complementary structural pattern. Tight views accounted for 0.680 of the graph derived attribution, whereas loose views accounted for 0.320. The larger tight contribution is consistent with the stronger performance effect observed for molecularly concordant neighborhoods in the module ablation analysis, while the nonzero loose attribution indicates that lower consistency PPI interactions still contribute complementary context. Overall, GNNExplainer shows that DRIVE predictions arise from gene dependent combinations of molecular alterations and local interaction structure rather than from a single uniform predictive signature.

**Supplementary Fig. S10 |** **GNNExplainer interpretation of representative candidate driver genes prioritized by DRIVE. a,b,** Local explanations for RELA and FYN. Central nodes denote the target candidate genes and surrounding nodes denote the six directly connected PPI neighbors with the highest GNNExplainer edge attribution. Edge width and annotated values indicate normalized edge attribution scores. Insets show normalized GNNExplainer feature attribution grouped into mutation frequency (MF), copy number aberration (CNA), DNA methylation (METH) and gene expression (GE). Driver scores denote the probabilities produced by DRIVE. **c,** Gene specific normalized omics attribution for RELA, FYN, ANLN and NR3C1. Each stacked bar sums to one. **d,** Global explanation summary across the PCDG set. Left, mean normalized omics attribution across candidate genes. Right, relative structural attribution assigned to tight and loose graph views. Structural values are normalized within graph derived edge attribution. GNNExplainer attribution reflects the predictive evidence used by DRIVE.

### S9 The unique candidates identified by DRIVE

Under the matched candidate lists used for this comparison, DRIVE retained 29 candidate genes that were absent from the candidate sets of EMOGI, deepCDG, SGCD, TREE, and MNDGNN, including BMP2, BMP4, BMP7, HDAC3, HDAC6, IGF2, IKBKG, INSR, IRS2, JUP, KAT5, PLEC, MEF2C, NFKBIA, PAK2, PDPK1, PIK3R2, PIK3R3, PLCG2, PPP2CA, PRKCQ, PRKCZ, PTPN1, SMAD7, TGFB2, TRAF6, WNT1, WNT2, YAP1.

Importantly, 10 of these DRIVE-specific candidates were already represented in our biological validation analyses, providing direct functional or pathway-level support. HDAC3, KAT5, and PPP2CA were included in the DepMap CRISPR analysis in Fig. 5c. HDAC3 is a class I histone deacetylase involved in chromatin regulation, cell-cycle control, and DNA-damage responses; KAT5, also known as TIP60, is a lysine acetyltransferase with established roles in chromatin remodeling, ATM activation, DNA double-strand break repair, and p53 regulation; and PPP2CA encodes the catalytic subunit of PP2A, a major phosphatase controlling PI3K/AKT, MAPK, and cell-cycle signaling. Their broad CRISPR dependencies across tumor cell lines provide direct functional support for their prioritization. Seven additional DRIVE-specific candidates, IKBKG, NFKBIA, PIK3R2, PIK3R3, PLCG2, PRKCQ, and TRAF6, were represented in the immune analyses in Fig. 6. IKBKG and NFKBIA are central regulators of canonical NF-κB signaling; PIK3R2 and PIK3R3 encode regulatory components of class I PI3K signaling that connect growth, survival, and immune-receptor pathways; PLCG2 and PRKCQ are important mediators of immune-receptor and T-cell activation signaling; and TRAF6 is a signaling adaptor and E3 ubiquitin ligase linking Toll-like receptor, cytokine-receptor, NF-κB, and MAPK pathways. Consistent with these functions, these genes were distributed across checkpoint regulation, T-cell receptor signaling, cytokine and chemokine signaling, and innate inflammatory programs in Fig. 6, showing that several candidates uniquely retained by DRIVE under the present comparison also receive orthogonal biological support from functional dependency and immune-regulatory analyses.

The remaining 19 DRIVE-specific candidates were not displayed in Figs. 5 and 6, but many belong to well-established cancer-associated signaling and regulatory systems. BMP2, BMP4, BMP7, TGFB2, and SMAD7 form a coherent TGF-β/BMP regulatory group involved in differentiation, epithelial-mesenchymal transition, invasion, and tumor-microenvironment regulation. WNT1, WNT2, and YAP1 connect WNT and Hippo signaling to proliferation, stemness, metastatic behavior, and therapeutic resistance. PDPK1 and PRKCZ participate in PI3K-dependent AGC-kinase signaling and cellular survival programs, while IGF2, IRS2 link growth-factor and metabolic signaling to PI3K/AKT, JAK/STAT, and related oncogenic processes. HDAC6 regulates protein acetylation, cytoskeletal dynamics, proteostasis, migration, and stress responses; PAK2 links RAC/CDC42 signaling to cytoskeletal remodeling and cell survival; PTPN1 regulates receptor tyrosine kinase, JAK/STAT, and metabolic signaling; JUP contributes to adherens junction and desmosomal organization and has established connections to cell adhesion and invasion; and MEF2C is a transcriptional regulator with important functions in hematopoietic and immune-cell programs and documented relevance to hematological malignancies. Taken together, the 29 DRIVE-specific candidates span multiple biologically coherent processes, including NF-κB inflammation, PI3K/AKT signaling, T-cell activation, TGF-β/BMP signaling, WNT/Hippo signaling, epigenetic regulation, DNA-damage responses, phosphorylation control, and cell adhesion.

These results indicate that DRIVE expands the candidate space beyond the representative comparison methods under the same candidate-selection framework while retaining candidates with substantial functional and mechanistic plausibility.

### S10 Prior evidence stratification and comparative biological characterization of DRIVE candidates

To distinguish DRIVE candidates with previous cancer related evidence from candidates with limited prior annotation, we systematically characterized the 186 predicted candidate driver genes using complementary evidence from cancer gene resources, protein protein interaction networks, CRISPR dependency screens, and tumor somatic mutation profiles.

#### S10.1 Annotation of previously reported and provisionally unreported DRIVE candidates

To determine the prior reporting status of the 186 DRIVE candidates, we first compared them with four established cancer gene reference resources used in our external evaluation, including OncoKB, ONGene, NCG candidate cancer genes, and the Bailey et al. pan cancer driver gene set. A candidate was classified as previously reported when it was present in at least one of these four resources. Candidates absent from all four resources were classified as provisionally unreported.

Among the 186 DRIVE candidates, 105 genes were previously reported, accounting for 56.5% of the complete candidate set, whereas 81 genes were provisionally unreported, accounting for 43.5% (Supplementary Fig. S11). To further characterize existing cancer related evidence, we additionally queried CancerMine Driver, CancerMine Oncogene, CancerMine tumor suppressor gene annotations, CCGD Rank A, and DriverDBv5. These resources were treated as supporting evidence rather than criteria for redefining the two candidate groups.

**Supplementary Fig. S11 | Prior cancer evidence supports DRIVE PCDGs.** Prior evidence stratification of 186 DRIVE PCDGs using OncoKB, ONGene, NCG candidate cancer genes and the Bailey et al. driver gene set, identifying 105 previously reported and 81 provisionally unreported PCDGs. CancerMine, CCGD Rank A and DriverDBv5 provide additional annotations.

#### S10.2 PPI network proximity of DRIVE candidates to known cancer genes

To characterize the network context of the DRIVE candidates, we analyzed high confidence human protein protein interactions from STRING v12.0. Only interactions with a combined score of at least 700 were retained. NCG v6.0 known cancer genes that did not overlap with the 186 DRIVE candidates were used as the known cancer gene reference set.

Network analysis was performed from two complementary perspectives. First, direct interactions between the 105 previously reported DRIVE candidates and 81 provisionally unreported DRIVE candidates were examined to determine the extent to which candidates from the two groups occupied interconnected network neighborhoods. Second, for each evaluable gene, we calculated the proportion of its direct STRING neighbors that belonged to the NCG v6.0 known cancer gene set. This known cancer gene neighbor fraction was then compared among NCG v6.0 known cancer genes, previously reported DRIVE candidates, and provisionally unreported DRIVE candidates.

The two DRIVE candidate groups showed extensive connectivity in the high confidence STRING network, with 129 direct interactions observed between previously reported and provisionally unreported candidates. At the whole set level, the median known cancer gene neighbor fractions were 0.200 for previously reported DRIVE candidates and 0.143 for provisionally unreported DRIVE candidates. These results indicate that the provisionally unreported PCDGs, despite their limited prior annotation, remain substantially embedded within known cancer gene neighborhoods, providing independent network proximity support for their potential cancer relevance.

These results indicate that the 81 provisionally unreported DRIVE candidates generally occupy cancer relevant interaction neighborhoods and show network proximity patterns broadly comparable to those of the 105 previously reported DRIVE candidates.

#### S10.3 Comparison of CRISPR dependency

To evaluate the functional dependency profiles of DRIVE candidates, we analyzed genome scale CRISPR screening data from DepMap Public 23Q4 using Chronos gene effect scores. For each gene, a dependency event was defined as a Chronos score below −0.5 in a cancer cell model. The dependency breadth of each gene was calculated as the percentage of evaluable cancer models satisfying this criterion.

Dependency breadth was compared among NCG v6.0 known cancer genes, the 105 previously reported DRIVE candidates, and the 81 provisionally unreported DRIVE candidates. Group differences were evaluated using two-sided Mann Whitney U tests, followed by Benjamini Hochberg correction for multiple comparisons. Cliff's δ was additionally calculated to quantify the effect size between the two DRIVE candidate groups.

Their distributions were highly similar, with FDR = 0.917 and |Cliff's δ| = 0.017 (Supplementary Fig. S12 a). Both DRIVE candidate groups showed greater dependency breadth than the NCG v6.0 known cancer gene reference group, with FDR = 0.011 for previously reported candidates and FDR = 0.029 for provisionally unreported candidates.

Thus, the 81 provisionally unreported DRIVE candidates did show comparable cancer cell dependency compared with the 105 previously reported DRIVE candidates.

**Supplementary Fig. S12 | Comparative biological characterization of previously reported and provisionally unreported DRIVE PCDGs.** a, DepMap Public 23Q4 CRISPR dependency rate of previously reported and provisionally unreported PCDGs, defined as the percentage of evaluable cancer models with Chronos gene effect scores below −0.5. b, Maximum cancer type specific mutation prevalence of the two PCDG groups across 16 TCGA cancer types based on PanCancer MC3 nonsynonymous somatic mutations. Violin plots show gene level distributions, boxplots indicate the median and interquartile range, and points represent individual genes. P values were calculated using two-sided Mann Whitney U tests.

#### S10.4 Comparison of somatic mutation patterns across TCGA cancer types

To examine tumor genomic alteration patterns in the DRIVE candidate genes, we analyzed PASS nonsynonymous somatic mutations from TCGA PanCancer MC3 across the 16 cancer types included in the DRIVE study. For each gene and cancer type, the mutation positive patient fraction was calculated as the proportion of evaluable patients carrying at least one nonsynonymous somatic mutation in that gene. The maximum mutation positive patient fraction across the 16 cancer types was then used to summarize the strongest cancer type specific mutational signal for each gene.

The distributions of this metric were compared among NCG v6.0 known cancer genes, the 105 previously reported DRIVE candidates, and the 81 provisionally unreported DRIVE candidates. Pairwise comparisons were conducted using two-sided Mann Whitney U tests and corrected using the Benjamini Hochberg procedure.

The median maximum mutation positive patient fraction was 4.87% in the previously reported DRIVE candidates and 4.34% in the provisionally unreported DRIVE candidates (Supplementary Fig. S12 b). No significant differences were observed between NCG v6.0 known cancer genes and previously reported DRIVE candidates (FDR = 0.837), between NCG v6.0 known cancer genes and provisionally unreported DRIVE candidates (FDR = 0.747), or between the two DRIVE candidate groups (FDR = 0.689).

These results indicate that DRIVE candidate prioritization is not restricted to genes with exceptionally high mutation frequencies.

#### S10.5 Background mutability adjusted cancer type recurrence analysis

Raw mutation frequency can be influenced by intrinsic gene specific mutability. Genes with larger coding regions or elevated background mutation rates can accumulate more passenger mutations and may therefore appear recurrently mutated even in the absence of cancer specific selection. We therefore performed an additional recurrence analysis accounting for background mutability to provide a more stringent assessment of the molecular alteration patterns observed in the DRIVE candidates.

For each gene, recurrent somatic mutation signals were evaluated across the same 16 TCGA cancer types after accounting for background mutability. The resulting number of cancer types showing recurrence was compared among NCG v6.0 known cancer genes, the 105 previously reported DRIVE candidates, and the 81 provisionally unreported DRIVE candidates. Continuous group comparisons were performed using Mann Whitney U tests, followed by Benjamini Hochberg correction.

After background mutability adjustment, no significant differences in the number of cancer types showing recurrent mutation were observed among the three groups, with all pairwise FDR values ≥ 0.187. In particular, the recurrence distribution of the 81 provisionally unreported DRIVE candidates was similar to that of the 105 previously reported DRIVE candidates or NCG v6.0 known cancer genes.

These results indicate that the molecular alteration profiles of provisionally unreported DRIVE candidates remain broadly comparable to those of previously characterized cancer related genes after accounting for variation in background mutability.

### S11 Driver associated genomic evidence supporting DRIVE PCDGs

To evaluate genomic characteristics associated with cancer driver activity independently of network proximity and functional dependency, we analyzed the predefined set of 186 DRIVE predicted candidate driver genes using TCGA PanCancer MC3 somatic mutation data. The analyses focused on complementary genomic signals related to tumor evolution, including positive selection, cancer type specific recurrence after background mutability adjustment, recurrent mutation hotspot enrichment, and convergent genomic evidence for provisionally unreported candidates.

**Supplementary Fig. S13 | Driver associated genomic signals across the predefined 186 PCDGs,** including pooled dN/dS for positive selection, mutability adjusted recurrence relative to matched background expectations, and enrichment of recurrent mutation hotspots from Cancer Hotspots v3. Points indicate effect estimates and horizontal lines indicate 95% confidence intervals; the null value is 1. PCDG, predicted candidate driver gene

#### S11.1 Positive selection analysis

Positive selection was evaluated using the ratio of nonsynonymous to synonymous substitution rates, dN/dS. Uniformly processed TCGA PanCancer MC3 somatic mutation data were used. 65 extremely hypermutated samples were excluded, leaving 5,856 tumors for background estimation. To reduce confounding caused by intrinsic differences in gene mutability, the background mutation model was estimated across the exome while accounting for trinucleotide mutational context and gene specific mutation rates.

Across the predefined 186 DRIVE PCDGs, the pooled dN/dS was 1.104 (95% CI, 1.066 to 1.143; P = 1.73 × 10⁻⁸; Supplementary Fig. S13). This result supports positive selection affecting the candidate set during tumor evolution.

We additionally evaluated the robustness of this signal across cancer types. Cancer type stratified random effects analysis yielded a dN/dS of 1.111 (95% CI, 1.070 to 1.153; P = 3.31 × 10⁻⁸; I² = 7.77%), indicating low between cancer heterogeneity and showing that the pooled signal was not dominated by a single tumor type. Because adjacent SNVs can arise from linked mutational events and potentially inflate substitution counts, the analysis was repeated after removing adjacent SNVs. The positive selection signal remained stable (dN/dS = 1.102; P = 3.65 × 10⁻⁸). These sensitivity analyses support the robustness of the positive selection signal across cancer contexts and mutation filtering strategies.

#### S11.2 Cancer type specific recurrence after background mutability adjustment

We next evaluated recurrent somatic mutation while explicitly controlling for differences in background gene mutability. For each DRIVE candidate within each cancer type, 50 background genes were matched according to synonymous mutation burden and nonsynonymous mutation burden outside the target cancer type. This matching strategy was used to construct a background expectation for genes with comparable intrinsic mutational propensity.

For each cancer type, the observed number of recurrently mutated DRIVE candidates was compared with the corresponding matched background expectation. Candidate recurrence exceeded the matched expectation in 13 of the 16 evaluated cancer types. Across the 16 cancer types, the median observed to expected ratio was 1.209 (95% CI, 1.065 to 1.276; P = 8.39 × 10⁻⁴; Supplementary Fig. S13). Thus, DRIVE PCDGs showed recurrent nonsynonymous mutation across multiple cancer types at levels exceeding those expected for genes with comparable background mutability.

#### S11.3 Recurrent mutation hotspot enrichment

To examine the functional localization of recurrent somatic alterations, we annotated established recurrent mutation hotspots using Cancer Hotspots v3. Hotspot containing genes were significantly enriched among DRIVE PCDGs, with an odds ratio of 3.698 (95% CI, 1.801 to 7.596; P = 0.00218; Supplementary Fig. S13).

This approximately 3.7 fold enrichment indicates that somatic mutations affecting DRIVE PCDGs preferentially include amino acid positions or functional regions with established evidence of recurrent mutation. This analysis therefore provides a complementary genomic signal of functional selection at recurrent mutation sites.

#### S11.4 Convergent genomic evidence for provisionally unreported PCDGs

We further evaluated the 81 provisionally unreported PCDGs using evidence independent of the cancer gene annotations employed to define their prior reporting status. All such database annotations were excluded from this prioritization analysis. A prespecified evidence rule was then applied using three genomic evidence layers: mutability matched recurrence in TCGA, replication in an independent patient cohort, and approximate clonality. A candidate was retained when at least two of these three evidence layers provided support.

Eleven provisionally unreported PCDGs satisfied this criterion: HSPA8, RFX5, VCL, UBR4, BTRC, TERF1, TLN1, PRPF8, USP34, HOXA5, and ANXA1. These genes therefore receive convergent support from at least two genomic evidence layers without using the annotations that defined their prior reporting status. This subset provides a focused group of previously under annotated candidates for subsequent functional and experimental validation.

### S12 Immunotherapy-related material

To evaluate the immunotherapy relevance of predicted candidate driver genes, we curated immunotherapy-related pathways from KEGG, Reactome and PID and grouped them into four mechanistic modules: checkpoint regulation and antigen presentation, T cell activation and cytotoxicity, IFN, cytokine and chemokine signaling, and innate inflammatory signaling. These modules were used to organize the pathway support map shown in Fig. 6a.

For each pathway $p$, let $G_{p}$ denote the genes annotated to pathway $p$, and let $G_{\mathrm{PCDG}}$ denote the candidate gene set used for immune analysis. Pathway enrichment was evaluated using a Fisher exact test against the background genes that could be mapped to the corresponding pathway database. Multiple testing correction was performed using the Benjamini Hochberg procedure, and pathways with FDR<0.05 were retained.

To reduce redundancy among highly overlapping pathways, we calculated the Jaccard similarity between every pair of enriched pathways:

$$\begin{aligned} J\left( p_{i},p_{j} \right)=\frac{\left| G_{p_{i}}\cap G_{p_{j}} \right|}{\left| G_{p_{i}}\cup G_{p_{j}} \right|}\#\left( 23 \right) \end{aligned}$$

For pathway pairs with $J\left( p_{i},p_{j} \right)>0.50$ we retained the pathway with the lower FDR and clearer mechanistic annotation. The remaining pathways were used for Fig. 6a and downstream immune priority scoring. To quantify gene-level immune relevance, we used a leave one gene out strategy. For each candidate gene $g$ and pathway $p$, gene $g$ was temporarily removed from the candidate set, and pathway enrichment was recalculated. The contribution of gene $g$ to pathway $p$ was defined as:

$$\begin{aligned} C_{g,p}=I\left( g\in G_{p} \right)\times w_{p}\times\text{Count}_{p,-g}\times\left[ -\log_{10}\left( \max\left( \text{FDR}_{p,-g},{10}^{-50} \right) \right) \right]\times\log_{2}\left( 1+\text{FE}_{p,-g} \right)\#\left( 24 \right) \end{aligned}$$

where $I\left( g\in G_{p} \right)$ indicates if gene $g$ belongs to pathway $p$, $w_{p}$ denotes the predefined weight of the immune mechanistic module to which pathway $p$ belongs, $\text{Count}_{p,-g}$ denotes the number of remaining candidate genes in pathway $p$ after removing gene $g$, $\text{FDR}_{p,-g}$ denotes the recalculated FDR after removing gene $g$, and $\text{FE}_{p,-g}$ denotes the corresponding fold enrichment. Three gene-level immune scores were then calculated:

$$\begin{aligned} \text{PathwayEvidence}_{g}=\sum_{p\in P_{\text{immune}}} C_{g,p}\#\left( 25 \right) \end{aligned}$$

$$\begin{aligned} \mathrm{MechanismBreadth}_{g}=\sum_{m\in M} I\left( \sum_{p\in m} C_{g,p}>0 \right)\#\left( 26 \right) \end{aligned}$$

$$\begin{aligned} \mathrm{CoreMechanism}_{g}=\sum_{p\in P_{\mathrm{core}}} C_{g,p}\#\left( 27 \right) \end{aligned}$$

where $P_{\mathrm{immune}}$ denotes all curated immunotherapy-related pathways, $M$ denotes the four immune mechanistic modules, and $P_{\mathrm{core}}$ denotes core immunotherapy-related pathways, including PD L1/PD 1 checkpoint, CTLA4 co-inhibition, T cell receptor signaling, natural killer cell-mediated cytotoxicity, interferon gamma signaling and MHC-mediated antigen presentation. Each of the three scores was standardized across candidate genes using z-score normalization. The final immune priority score was defined as

$$\begin{aligned} \mathrm{ImmunePriorityScore}_{g}=0.65z\left( \mathrm{PathwayEvidence}_{g} \right) \\ +0.20z\left( \mathrm{MechanismBreadth}_{g} \right)+0.15z\left( \mathrm{CoreMechanism}_{g} \right) \#\left( 28 \right) \end{aligned}$$

Genes with higher ImmunePriorityScore values were considered to have stronger and broader support from immunotherapy-related pathways. In Fig. 6a, candidate genes were ordered according to this score, and pathway-level enrichment was summarized using gene ratio, overlap count and FDR. **Table S7** provides the complete gene-level immune evidence used for Fig. 6a, including ImmunePriorityScore, PathwayEvidence, MechanismBreadth, CoreMechanism, significant immune evidence modules, supported modules, representative supporting pathway terms, significant pathway terms and top-gene selection indicators. The top 20 genes used for the pathway support map in Fig. 6a were selected according to ImmunePriorityScore.

### S13 Sensitivity analysis of representative-gene prioritization and random-gene-set controls

#### S13.1 Rationale and predefined prediction-score-based selection

The biological analyses in Fig. 6 were designed to characterize immune-related and pharmacogenomic features of DRIVE-predicted candidate driver genes. The pathway-level enrichment analysis used the complete PCDG set, whereas representative genes shown in the pathway-support map and immune-network visualization were prioritized according to ImmunePriorityScore.

To evaluate the robustness of the biological interpretation to representative-gene selection, we therefore performed an additional sensitivity analysis in which the selection criterion was defined independently of all downstream immune and pharmacogenomic information.

All 186 PCDGs were ranked in descending order according to their DRIVE prediction probabilities. The 20 highest-scoring genes were selected before examining ImmunePriorityScore, immune annotations, pathway membership, immune-network information, or GDSC drug-response associations. The resulting prediction-score-prioritized Top 20 genes were:

SOS1, NFKBIA, CTNNA1, YAP1, FANCM, NEDD4, FYN, NR3C1, NFKB1, SPI1, IRAK1, RELA, LYN, BPTF, TGFBR1, PTPRJ, KMT2B, FOXM1, SOX10, and CHEK1.

Only five genes, SOS1, NFKBIA, FYN, NFKB1, and RELA, overlapped with the original ImmunePriorityScore-based Top 20. Thus, 75% of the representative genes changed when immune-related information was excluded from the selection procedure.

The same downstream analyses used for Fig. 6 were subsequently repeated using the prediction-score-prioritized genes. The pathway definitions, immune-network evidence sources and scoring rules, GDSC pharmacogenomic data, statistical procedures, and significance thresholds were retained without re-optimization.

#### S13.2 Sensitivity analysis using prediction-score-prioritized genes

**S13.2.1 Immunotherapy-related pathway convergence**

The immunotherapy-related pathway analysis continued to identify multiple immune-associated programs under the prediction-score-based selection criterion (Supplementary Fig. S14a).

Among the predefined immune pathways, 10 reached FDR<0.05. These included PD-L1 expression and PD-1 checkpoint signaling, CTLA4-associated co-inhibition, T-cell receptor signaling, natural killer cell-mediated cytotoxicity, interferon-related signaling, cytokine and chemokine signaling, Toll-like receptor cascades, and NF-κB signaling.

Compared with the original ImmunePriorityScore-based visualization, pathway support was more selectively distributed across the prediction-prioritized genes. This reduction was expected because immune-related information no longer contributed to representative-gene selection. Nevertheless, the major immune programs observed in the original analysis remained detectable among genes prioritized solely according to DRIVE prediction probability.

**S13.2.2 Evidence-weighted immune-network connectivity**

We next reconstructed the immune-regulatory network using representative genes from the prediction-score-prioritized set and the same predefined immune reference nodes, evidence sources, edge-retention criteria, and weighting procedure used in the original Fig. 6 analysis (Supplementary Fig. S14b).

The resulting network retained connections between prediction-prioritized PCDGs and six predefined immune functional modules: immune checkpoint; antigen presentation; cytokine and chemokine signaling; cytotoxic effector function; T-cell activation; and innate inflammatory signaling.

For visualization clarity, representative genes from the highest-ranked portion of the prediction-score-prioritized set were displayed as inner candidate nodes, whereas immune reference nodes were arranged according to their predefined functional modules.

Using the same evidence-weighted network scoring procedure applied in the random-set analysis, the prediction-score-prioritized gene set produced an observed connectivity score of 121.

**S13.2.3 Pharmacogenomic associations**

The GDSC drug-sensitivity analysis was repeated using the prediction-score-prioritized genes without changing the pharmacogenomic dataset, correlation definition, multiple-testing procedure, or FDR threshold (Supplementary Fig. S14c).

Significant positive and negative correlations between candidate-gene expression and drug-response phenotypes remained broadly distributed across multiple candidates and compounds. In total, 340 gene-drug associations reached FDR<0.05.

Thus, extensive pharmacogenomic associations remained detectable when representative candidates were selected solely according to DRIVE prediction probability and without reference to downstream immune or drug-response information.

#### S13.3 Random-gene-set permutation analysis

To quantify the observed pathway, network, and pharmacogenomic patterns relative to alternative gene subsets from the same DRIVE candidate pool, we performed a permutation-based random-gene-set analysis.

Let $C$ denote the fixed set of 186 DRIVE PCDGs. For permutation $b$, a random subset $R_{b}$ containing 20 genes was sampled without replacement: $R_{b}\subset C, \left| R_{b} \right|=20.$ This procedure was repeated $B=1,000$ times.

Each random gene set was processed using exactly the same pathway analysis, immune-network scoring procedure, GDSC analysis, statistical thresholds, and multiple-testing procedures used for the prediction-score-prioritized Top 20. No analysis parameter was re-estimated or optimized during the permutation procedure.

Restricting the randomization background to the same 186-PCDG pool ensured that both the observed and random sets had already satisfied the DRIVE candidate-selection criterion. The random-set analysis therefore evaluated the additional concentration of biological evidence associated with prediction-probability-based prioritization within the DRIVE candidate universe.

Three summary statistics were specified before comparison with the null distributions:

$T_{\text{path}}=$ number of significant predefined immune pathways, $T_{\text{network}}=$ evidence-weighted immune-network connectivity score, and $T_{\text{drug}}=$ number of significant GDSC gene-drug associations. For each statistic $T$, the one-sided empirical permutation P value was calculated as

$$\begin{aligned} P_{\text{emp}}=\frac{1+\sum_{b=1}^{B} I\left( T_{b}\geq T_{\text{obs}} \right)}{B+1}\#\left( 29 \right) \end{aligned}$$

where $T_{\text{obs}}$ represents the statistic obtained from the prediction-score-prioritized Top 20, $T_{b}$ represents the corresponding statistic obtained from permutation $b$, and $I\left( \cdot\right)$ denotes the indicator function. The finite-permutation correction prevents zero empirical P values.

**Supplementary Fig. S14 | Sensitivity analysis using PCDGs prioritized solely by DRIVE prediction probability. a,** Immunotherapy-related pathway programs and supporting candidate genes for the 20 PCDGs with the highest DRIVE prediction probabilities. Gene selection was performed before examining ImmunePriorityScore, immune annotations, pathway membership, immune-network information, or drug-response data. Columns indicate candidates ordered by DRIVE prediction probability. Dots indicate candidate support for the corresponding predefined immune pathway. The pathway summary on the right report’s gene ratio, overlapping gene count, and FDR-adjusted enrichment significance. **b,** Evidence-weighted immune-regulatory network reconstructed using representative genes from the prediction-score-prioritized set. Inner purple nodes denote DRIVE-prioritized candidate genes and outer nodes denote predefined immune reference genes grouped into immune checkpoint, antigen presentation, cytokine and chemokine signaling, cytotoxic effector function, T-cell activation, and innate inflammatory signaling. Edge width indicates the amount of shared pathway or module evidence supporting the corresponding connection. **c,** Associations between mRNA expression of prediction-score-prioritized PCDGs and GDSC drug-response phenotypes. Dot color indicates the direction and magnitude of the correlation, dot size indicates $-\log_{10} (FDR)$, and outlined dots indicate associations reaching FDR≤0.05.

#### S13.4 Comparison with random-gene-set distributions

**S13.4.1 Immune-pathway convergence**

The prediction-score-prioritized Top 20 contained 10 significant predefined immune pathways.

Among the 1,000 random 20-gene sets sampled from the same PCDG pool, only 14 reached or exceeded 10 significant pathways.

The corresponding empirical permutation P value was 0.015, The observed pathway-convergence statistic was therefore located in the upper tail of the random-set distribution (Supplementary Fig. S15a).

**S13.4.2 Immune-network connectivity**

The prediction-score-prioritized genes produced an evidence-weighted immune-network connectivity score of 121.

Only 20 of the 1,000 random sets reached or exceeded this value, corresponding to 0.021, Thus, the network connectivity observed among prediction-prioritized candidates was also located in the upper tail of the corresponding empirical null distribution (Supplementary Fig. S15b).

**S13.4.3 Pharmacogenomic associations**

The prediction-score-prioritized genes yielded 340 significant GDSC gene-drug associations.

Only 9 of the 1,000 random sets reached or exceeded this value, corresponding to 0.010, Among the three evaluated endpoints, the pharmacogenomic statistic showed the strongest separation from its corresponding random-set distribution (Supplementary Fig. S15c).

#### S13.5 Interpretation of the sensitivity and random-set analyses

Together, these analyses directly evaluate the potential influence of post hoc representative-gene prioritization in Fig. 6.

First, the prediction-score-based sensitivity analysis used a gene-selection criterion determined exclusively by DRIVE model output and independent of downstream immune and pharmacogenomic annotations. Only five of the 20 genes overlapped with the original ImmunePriorityScore-based Top 20, yet major immune-pathway programs, immune-network connections, and extensive GDSC associations remained detectable.

Second, the observed pathway-convergence, network-connectivity, and pharmacogenomic-association statistics all exceeded the corresponding distributions generated from 1,000 random 20-gene subsets of the same 186-PCDG candidate pool.

These results support the robustness of the principal Fig. 6 biological patterns to representative-gene selection and reduce the likelihood that the observations arise solely from selecting candidates according to immune-derived annotations.

**Supplementary Fig. S15 | Random-gene-set controls for the prediction-score-based sensitivity analysis.** Twenty genes were randomly sampled without replacement from the fixed set of 186 DRIVE PCDGs. Each random gene set was evaluated using the same pathway, immune-network, and pharmacogenomic procedures and significance thresholds applied to the prediction-score-prioritized Top 20. The procedure was repeated 1,000 times. Histograms show the resulting empirical null distributions, and dashed vertical lines indicate the observed statistics for the prediction-score-prioritized genes. **a,** Distribution of the number of significant predefined immune pathways. The observed value was 10. Fourteen of 1,000 random gene sets reached or exceeded this value, corresponding to an empirical P=0.015. **b,** Distribution of the evidence-weighted immune-network connectivity score. The observed value was 121. Twenty of 1,000 random gene sets reached or exceeded this value, corresponding to an empirical P=0.021. **c,** Distribution of the number of significant GDSC gene-drug associations. The observed value was 340. Nine of 1,000 random gene sets reached or exceeded this value, corresponding to an empirical P=0.010.

### S14 Covariate adjusted immune associations and clinical relevance of DRIVE PCDGs

To evaluate the robustness and clinical relevance of immune associations involving DRIVE predicted candidate driver genes (PCDGs), we extended the analysis by performing cancer type specific covariate adjustment, controlling expression collinearity, evaluating predefined DRIVE immune signatures in independent immunotherapy cohorts (Supplementary Table S8), and examining their associations with overall survival.

#### S14.1 Cancer type specific associations between DRIVE PCDGs and LM22 immune cell states

To determine the extent to which the immune associations of DRIVE PCDGs were reproducible within cancer types and to characterize their immune cell specificity, we evaluated the predefined set of 186 DRIVE PCDGs separately within 16 TCGA cancer types, including BLCA, BRCA, CHOL, COAD, ESCA, HNSC, KIRC, KIRP, LIHC, LUAD, LUSC, PAAD, PRAD, READ, THCA, and UCEC. Within each cancer type, we examined associations between PCDG expression and the 22 CIBERSORT LM22 immune cell states.

All analyses were performed separately within individual cancer types, thereby controlling differences in cancer type composition by design. The resulting associations were widely distributed across cancer types and LM22 cell states, while their magnitude and direction varied substantially among cancers and immune cell populations (Fig. S16). The number of unique PCDGs associated with at least one LM22 immune cell state ranged from 54 in CHOL to 182 in THCA, with 177 in KIRC, 175 in BRCA, 166 in LUAD, and 161 in HNSC. Thus, the immune associations involved substantial fractions of the 186 PCDGs in multiple cancer types, while their distributions across genes and LM22 phenotypes remained strongly cancer type dependent.

The heatmap in Fig. S16 summarizes both the number and direction of significant associations for each cancer type and LM22 cell state. Cell values indicate the number of associated PCDGs, while the color scale represents the median covariate adjusted partial correlation among the corresponding genes. The right hand bars show the total number of unique PCDGs associated with at least one LM22 cell state within each cancer type. These results support heterogeneous and cancer type specific associations between DRIVE PCDGs and LM22 immune cell states.

**Supplementary Fig. S16 | Cancer type specific associations between DRIVE PCDGs and the 22 CIBERSORT LM22 immune cell states after covariate adjustment.** Cell values indicate the number of associated PCDGs, cell color represents the median adjusted partial correlation, and bars on the right indicate the number of unique PCDGs associated with at least one LM22 cell state in each cancer type.

#### S14.2 Robustness to covariate adjustment and expression collinearity

We next tested the robustness of these within cancer associations after accounting for major biological, genomic, technical, and expression related confounding factors. The partial correlation models adjusted for tumor purity, tumor mutational burden, copy number variation burden, and available sequencing platform or technical batch factors.

Across all evaluable gene and immune cell associations, the overall effect structure remained highly consistent before and after covariate adjustment, with a Pearson correlation of 0.880 between the unadjusted correlations and covariate adjusted partial correlations (Fig. 6b). At the same time, covariate adjustment materially affected the statistical classification of a subset of associations. Specifically, 8,533 associations remained significant after adjustment and were classified as retained, 5,782 were significant before adjustment but no longer significant after adjustment and were classified as attenuated, and 1,522 reached significances only after adjustment and were classified as emergent. The remaining 48,579 associations did not reach the significance criterion either before or after adjustment.

The high concordance of effect sizes indicates that the overall gene immune association structure was largely preserved after confounder control. The 5,782 attenuated and 1,522 emergent associations further demonstrate that adjustment meaningfully refined individual association calls rather than leaving all associations unchanged. We therefore based all subsequent immune analyses on the covariate adjusted results.

Collectively, after controlling for tumor purity, tumor mutational burden, copy number variation, cancer type differences, batch effects, and expression collinearity, DRIVE PCDGs retained widespread associations with LM22 immune cell states, with substantial heterogeneity across cancer types, genes, and immune cell populations.

#### S14.3 Association of predefined DRIVE immune signatures with immunotherapy clinical benefit

We next examined the clinical relevance of these adjusted immune associations using multiple independent immunotherapy cohorts representing KIRC, LUAD, HNSC, ESCA, CHOL, BLCA, LIHC, BRCA, and PAAD.

For each cancer type, the DRIVE immune signature and its effect direction were predefined from the covariate adjusted TCGA analysis and then applied to the corresponding cancer type matched immunotherapy cohort. The signatures were therefore fixed before evaluation of immunotherapy clinical outcomes.

Across all cohorts, the covariate adjusted clinical benefit effect estimates were positive, indicating that the higher benefit or responder groups consistently tended to show higher scores for the predefined DRIVE immune signatures (Fig. S17). The magnitude and uncertainty of the individual cohort estimates varied, consistent with differences in cancer type, cohort size, and treatment setting.

Random effects meta analysis yielded an overall difference of 0.50 SD, with a 95% CI of 0.24 to 0.76, P = 1.50 × 10⁻⁴, and I² = 32%. The positive pooled estimate between cohort heterogeneity indicate a directionally consistent association between the predefined DRIVE immune signatures and immunotherapy clinical benefit across the all-independent cancer type matched cohorts.

These results provide an independent clinical extension of the TCGA immune association analysis. They support an association between the covariate adjusted DRIVE immune signatures and clinical benefit from immune checkpoint inhibitor treatment across multiple cancer contexts.

**Supplementary Fig. S17 | Association of predefined DRIVE immune signatures with immunotherapy clinical benefit across nine independent cancer type matched cohorts.** Points and horizontal lines indicate adjusted standardized mean differences and 95% CIs. The diamond indicates the random effects summary estimate.

#### S14.4 Association of DRIVE immune signatures with overall survival

As an additional clinical endpoint, we examined associations between the cancer type specific DRIVE immune signatures and overall survival using covariate adjusted multivariable Cox proportional hazards models. Survival analyses were performed independently within 10 TCGA cancer types (Fig. S18).

Covariate adjusted multivariable Cox analyses across 10 TCGA cancer types showed a broadly consistent direction of survival association. The combined evidence across cancer types was highly significant (Fisher combined P = 4.45 × 10⁻¹⁰), supporting a robust overall association between the DRIVE immune signatures and overall survival across multiple cancer contexts. KIRC showed one of the strongest cancer type specific associations, with an HR of 1.51 (95% CI, 1.28 to 1.77; FDR = 7.15 × 10⁻⁶). These results provide independent clinical evidence linking the cancer type specific immune associations of DRIVE PCDGs to patient survival.

These results provide an additional clinical association endpoint and show that a subset of cancer type specific DRIVE immune signatures is associated with overall survival. The observed survival effects were cancer type dependent, consistent with the heterogeneity observed in the LM22 immune association analysis.

Overall, the analyses show that DRIVE PCDGs retain widespread and cancer type specific associations with LM22 immune cell states after controlling for tumor purity, tumor mutational burden, copy number variation, cancer type differences, batch effects, and expression collinearity. Covariate adjusted multivariable Cox analyses across 10 TCGA cancer types showed a broadly consistent direction of survival association. These analyses support statistical associations of DRIVE PCDGs with cancer type specific immune infiltration, immunotherapy clinical benefit, and overall survival and provide candidate evidence for subsequent functional investigation.

**Supplementary Fig. S18 | Covariate adjusted associations between cancer type specific DRIVE immune signatures and overall survival across 10 TCGA cancer types.** Points and horizontal lines indicate hazard ratios and 95% CIs from multivariable Cox models. FDR adjusted P values are reported for each cancer type.

### S15 Feature and CKA similarity equations

To quantify the representation-level effects of DRIVE, we performed feature similarity offset and centered kernel alignment (CKA) analyses. Feature similarity offset was used to evaluate local similarity changes from the raw feature space to the learned representation space. CKA was used to measure global representational similarity among raw omics features and learned omics representations.

For feature similarity offset, we compared cosine similarity before and after representation learning. For gene $v_{i}$, its PPI neighbor $v_{j}\in\mathcal{N}_{i}$, and omics type $m$, raw feature similarity was calculated as:

$$\begin{aligned} s_{ij}^{f,m}=\frac{\left( \mathbf{f}_{i}^{m} \right)^{\top}\mathbf{f}_{j}^{m}}{\parallel\mathbf{f}_{i}^{m}\parallel_{2}\parallel\mathbf{f}_{j}^{m}\parallel_{2}}\#\left( 30 \right) \end{aligned}$$

and learned representation similarity was calculated as:

$$\begin{aligned} s_{ij}^{z,m}=\frac{\left( \mathbf{z}_{i,s}^{m} \right)^{\top}\mathbf{z}_{j,s}^{m}}{\parallel\mathbf{z}_{i,s}^{m}\parallel_{2}\parallel\mathbf{z}_{j,s}^{m}\parallel_{2}}\#\left( 31 \right) \end{aligned}$$

The edge level feature similarity offset was defined as:

$$\begin{aligned} \Delta s_{ij}^{m}=s_{ij}^{z,m}-s_{ij}^{f,m}\#\left( 32 \right) \end{aligned}$$

For each gene, the gene level offset was obtained by averaging over its PPI neighbors:

$$\begin{aligned} \Delta s_{i}^{m}=\frac{1}{\left| \mathcal{N}_{i} \right|}\sum_{v_{j}\in\mathcal{N}_{i}} \Delta s_{ij}^{m}\#\left( 33 \right) \end{aligned}$$

Driver and non-driver offsets were then computed as

$$\begin{aligned} \overline{\Delta s}_{+}^{m}=\frac{1}{\left| V_{L}^{+} \right|}\sum_{v_{i}\in V_{L}^{+}} \Delta s_{i}^{m}, \overline{\Delta s}_{-}^{m}=\frac{1}{\left| V_{L}^{-} \right|}\sum_{v_{i}\in V_{L}^{-}} \Delta s_{i}^{m} \#\left( 34 \right) \end{aligned}$$

where $V_{L}^{+}$ and $V_{L}^{-}$ denote labeled driver and non-driver genes. The class level offset difference was defined as:

$$\begin{aligned} \Delta_{\text{class}}^{m}=\overline{\Delta s}_{+}^{m}-\overline{\Delta s}_{-}^{m}\#\left( 35 \right) \end{aligned}$$

A positive $\Delta_{\text{class}}^{m}$ indicates that DRIVE increases local neighborhood similarity more strongly for driver genes than for non-driver genes.

For omics type $m\in\{\text{MF},\text{CNA},\text{GE},\text{METH}\}$, let $\mathbf{F}^{m}=\left[ \mathbf{f}_{1}^{m},\mathbf{f}_{2}^{m},\ldots,\mathbf{f}_{N}^{m} \right]^{\top}\in\mathbb{R}^{N\times16}$ denote the raw omics feature matrix. Let $\mathbf{Z}_{s}^{m}=\left[ \mathbf{z}_{1,s}^{m},\mathbf{z}_{2,s}^{m},\ldots,\mathbf{z}_{N,s}^{m} \right]^{\top}\in\mathbb{R}^{N\times d}$ denote the learned omics-specific representation matrix, and let $\mathbf{Z}_{c}=\left[ \mathbf{z}_{1,c},\mathbf{z}_{2,c},\ldots,\mathbf{z}_{N,c} \right]^{\top}\in\mathbb{R}^{N\times d}$ denote the learned omics-common representation matrix. For two centered matrices $\mathbf{X}$ and $\mathbf{Y}$, linear CKA was calculated as:

$$\begin{aligned} \mathrm{CKA}\left( \mathbf{X},\mathbf{Y} \right)=\frac{\parallel\mathbf{X}^{\top}\mathbf{Y}\parallel_{F}^{2}}{\parallel\mathbf{X}^{\top}\mathbf{X}\parallel_{F}\parallel\mathbf{Y}^{\top}\mathbf{Y}\parallel_{F}}\#\left( 36 \right) \end{aligned}$$

where $\parallel\cdot\parallel_{F}^{2}$ denotes the Frobenius norm. Higher CKA values indicate stronger representational similarity. We calculated CKA among raw omics features, between raw omics features and learned omics specific representations, and between raw omics features and the omics common representation. CKA values were first computed for each PPI network and then averaged across the six benchmark networks.

Together, CKA and feature similarity offset analyses show that DRIVE reduces cross omics redundancy, preserves omics specific information and reshapes raw omics features into more discriminative learned representations.

### S16 Network and CRISPR analysis of PCDGs

To evaluate the biological relevance of predicted candidate driver genes (PCDGs), we performed PPI proximity analysis with known driver genes (KDGs) and CRISPR dependency analysis using DepMap gene effect scores.

For Fig. 5a, each gene was assigned two ranks. The driver score rank was obtained by sorting all genes in descending order according to the driver score predicted by DRIVE using Eq. 46. The KDG PPI rank was obtained by sorting all genes in descending order according to the number of one hop PPI interactions with KDGs. Spearman correlation was used to quantify the association between these two rankings.

For Fig. 5b, we calculated the fraction of PPI interactions involving KDGs for each gene. Let $V$ denote all genes, $V_{L}^{+}$ denote KDGs, $P$ denote PCDGs, and $B=V/P$ denote background genes. For gene $g$, the KDG interaction fraction was defined as

$$\begin{aligned} f_{KDGs}\left( g \right)=\frac{|N\left( g \right)\cap V_{L}^{+}|}{N\left( g \right)}\#\left( 37 \right) \end{aligned}$$

where $N\left( g \right)$ denotes the one hop PPI neighbors of gene $g$. Genes without PPI neighbors were excluded. The empirical cumulative distribution functions of PCDGs and background genes were calculated as:

$$\begin{aligned} F_{P}\left( t \right)=\frac{1}{|P|}\sum_{g\in P} \mathbf{1}(f_{KDGs}\left( g \right)\leq t)\#\left( 38 \right) \end{aligned}$$

$$\begin{aligned} F_{B}\left( t \right)=\frac{1}{|B|}\sum_{g\in B} \mathbf{1}(f_{KDGs}\left( g \right)\leq t)\#\left( 39 \right) \end{aligned}$$

where $\boldsymbol{1}\left( \cdot\right)$ is the indicator function. A right shifted eCDF curve indicates a higher fraction of interactions with KDGs.

For the inset in Fig. 5b, we measured the interaction enrichment of each KDG with PCDGs and with all genes. For a known driver gene $g^{+}$, the red bar was calculated as:

$$\begin{aligned} redbar\left( g^{+} \right)=\frac{|g\in P:g\in N(g^{+})|}{|P|}\#\left( 40 \right) \end{aligned}$$

and the blue bar was calculated as:

$$\begin{aligned} bluebar\left( g^{+} \right)=\frac{|g\in V:g\in N(g^{+})|}{|V|}\#\left( 41 \right) \end{aligned}$$

The top KDGs in the inset were selected according to their interaction fractions with PCDGs.

For Fig. 5c, DepMap CRISPR gene effect scores were used to assess functional dependency of PCDGs across 1,086 tumor cell lines. Let $E_{g,c}$ denote the CRISPR gene effect score of gene $g$ in tumor cell line $c$. Lower values indicate stronger growth inhibition after CRISPR perturbation. A dependency event was defined as

$$\begin{aligned} D_{g,c}=\left\{ \begin{aligned} 1,E_{g,c}\leq-0.50 \\ 0,E_{g,c}>-0.50 \end{aligned} \right.\#\left( 42 \right) \end{aligned}$$

A gene was classified as CRISPR dependency supported if dependency events were observed in at least 10% of tumor cell lines, corresponding to at least 109 cell lines. Enrichment of CRISPR dependency supported genes in PCDGs was evaluated using Fisher exact test.

For Fig. 5c, the fraction of dependent tumor cell lines for gene $g$ was calculated as:

$$\begin{aligned} F_{dep}\left( g \right)=\frac{\sum_{c\in C} D_{g,c}}{|C|}\#\left( 43 \right) \end{aligned}$$

where $C$ denotes tumor cell lines with available DepMap gene effect scores for gene $g$. The minimum and mean gene effect scores were calculated as:

$$\begin{aligned} E_{g}^{min}=\min_{c\in C} E_{g,c}\#\left( 44 \right) \end{aligned}$$

$$\begin{aligned} E_{g}^{mean}=\frac{1}{|C|}\sum_{c\in C} E_{g,c}\#\left( 45 \right) \end{aligned}$$

Missing values were excluded from these calculations. In Fig. 5c, dot size represents $\boldsymbol{\mid}E_{g}^{mean}\boldsymbol{\mid}$, dot color represents the DRIVE prediction score, red points indicate $\boldsymbol{\mid}E_{g}^{min}\boldsymbol{\mid}$, and blue points indicate $\boldsymbol{\mid}E_{g}^{mean}\boldsymbol{\mid}$. Together, these analyses provide network level and perturbation-based evidence supporting the cancer relevance of PCDGs.

### S17 Detailed mathematical equation of DRIVE

This section provides the full mathematical equation of DRIVE that complements the concise model description in the main text. DRIVE is organized into three modules: dual-view disentangled graph attention, contrastive multi-omics fusion, and prediction with joint optimization. The main text retains the equations that define the principal methodological operations, whereas the implementation-level projections, attention normalization, auxiliary mappings and loss construction are detailed below.

#### S17.1 Problem formulation and omics-specific feature encoding

Cancer driver gene identification is formulated as semi-supervised node classification on an omics-level gene graph $G=\left( V,E,\mathbf{F} \right)$. Here, $V=\{v_{i}{\}}_{i=1}^{N}$ denotes the set of $N$ genes, $E\subseteq V\times V$ denotes protein-protein interactions, and $\mathbf{F}\in\mathbb{R}^{N\times M\times C}$ is the multi-omics feature tensor. $M$ is the number of omics types, $C$ is the number of cancer types, and $\mathcal{M=\{}mf,cna,meth,ge\}$ denotes mutation frequency, copy-number aberration, DNA methylation and gene expression. For gene $v_{i}$ and omics type $m\mathcal{\in M}$, $\mathbf{f}_{i}^{m}\in\mathbb{R}^{C}$ denotes its pan-cancer omics profile.

The node set contains labeled known drivers, labeled non-drivers and unlabeled genes. We denote the labeled set by $V_{L}$ and the unlabeled set by $V_{U}$. For compact notation in this section, $P_{L}$ denotes the labeled driver subset and $N_{L}$ denotes the labeled non-driver subset; these correspond to $V_{L}^{+}$ and $V_{L}^{-}$ in the main text, respectively. Given $G$, DRIVE learns a parameterised function $f_{\theta}\left( \cdot\right)$ and predicts a driver probability for every gene:

$$\begin{aligned} \hat{\mathbf{Y}}=f_{\theta}\left( G \right), \hat{\mathbf{Y}}\in\left[ 0,1 \right]^{N}\#\left( 46 \right) \end{aligned}$$

The element $\hat{y}_{i}\in\left[ 0,1 \right]$ represents the predicted probability that gene $v_{i}$ is a cancer driver gene. For unlabeled genes, higher probabilities indicate stronger computational evidence for candidate driver status.

Before graph-view construction, each omics branch independently maps the original $C$-dimensional profile into a $d$-dimensional latent space through a two-layer nonlinear encoder:

$$\begin{aligned} \mathbf{x}_{i}^{m}=\mathbf{W}_{2}^{m}\phi\left( \mathbf{W}_{1}^{m}\mathbf{f}_{i}^{m}+\mathbf{b}_{1}^{m} \right)+\mathbf{b}_{2}^{m}\#\left( 47 \right) \end{aligned}$$

Here, $\mathbf{W}_{1}^{m}\in\mathbb{R}^{d\times C}$ and $\mathbf{W}_{2}^{m}\in\mathbb{R}^{d\times d}$ are learnable parameter matrices, $\mathbf{b}_{1}^{m},\mathbf{b}_{2}^{m}\in\mathbb{R}^{d}$ are bias vectors, and $\phi\left( \cdot\right)$ denotes a nonlinear activation function. The resulting single-omics node embedding is $\mathbf{x}_{i}^{m}\in\mathbb{R}^{d}$, and the embedding matrix for omics type $m$ is denoted by $\mathbf{X}^{m}\in\mathbb{R}^{N\times d}$.

#### S17.2 Dynamic heterophily disentanglement and graph-view construction

For each PPI edge connecting genes $v_{i}$ and $v_{j}$, DRIVE evaluates omics-level embedding consistency using the cosine similarity defined in main text Eq. 1. The original edge set is then partitioned into tight and loose subsets according to the threshold rule in main text Eq. 2. These edge subsets define two attributed graph views for each omics type:

$$\begin{aligned} G_{m}^{tight}=\left( V,E_{m}^{tight},\mathbf{X}^{m} \right), G_{m}^{loose}=\left( V,E_{m}^{loose},\mathbf{X}^{m} \right)\#\left( 48 \right) \end{aligned}$$

Because $\mathbf{X}^{m}$ is updated jointly with the model parameters, the consistency scores and the associated edge assignments are recomputed during optimization. The graph views therefore evolve with the learned omics representation space. The tight view preferentially retains interactions between genes with concordant molecular profiles, whereas the loose view preserves lower-consistency interactions that can still provide complementary biological context in heterophilic PPI networks.

#### S17.3 View-oriented attention and omics-level representation learning

For each omics type $m$ and attention head $h\in\{1,\ldots,H\}$, DRIVE first projects the single-omics node embedding into query, key and value spaces:

$$\begin{aligned} \mathbf{q}_{i}^{m,h}=\mathbf{W}_{Q}^{m,h}\mathbf{x}_{i}^{m}, \mathbf{k}_{i}^{m,h}=\mathbf{W}_{K}^{m,h}\mathbf{x}_{i}^{m}, \mathbf{v}_{i}^{m,h}=\mathbf{W}_{V}^{m,h}\mathbf{x}_{i}^{m}\#\left( 49 \right) \end{aligned}$$

The projection matrices $\mathbf{W}_{Q}^{m,h},\mathbf{W}_{K}^{m,h},\mathbf{W}_{V}^{m,h}\in\mathbb{R}^{d/H\times d}$ are independently learned for each omics branch and attention head. For graph view $r\in\{tight,loose\}$ and neighbor $v_{j}\in\mathcal{N}_{i}^{m,r}$, a symmetric pairwise interaction context is constructed as

$$\begin{aligned} \mathbf{c}_{i,j}^{m,r,h}=\left[ \mathbf{q}_{i}^{m,h}\odot\mathbf{k}_{j}^{m,h} \| \mathbf{k}_{i}^{m,h}\odot\mathbf{q}_{j}^{m,h} \right]\#\left( 50 \right) \end{aligned}$$

Here, $\odot$ denotes element-wise multiplication and $\|$ denotes vector concatenation. The symmetric interaction encoding allows both directions of the pairwise gene association to contribute to attention estimation. The unnormalised coefficient is calculated as

$$\begin{aligned} \tilde{a}_{ij}^{m,r,h}=\phi\left( \left( \mathbf{a}^{m,r,h} \right)^{\top}\mathbf{c}_{i,j}^{m,r,h}+b^{m,r,h} \right)\#\left( 51 \right) \end{aligned}$$

where $\mathbf{a}^{m,r,h}\in\mathbb{R}^{2d/H}$ and $b^{m,r,h}\mathbb{\in R}$ are learnable parameters. Attention coefficients are normalised over the view-specific neighborhood:

$$\begin{aligned} a_{ij}^{m,r,h}=\frac{\exp\left( \tilde{a}_{ij}^{m,r,h} \right)}{\sum_{v_{j'}\in\mathcal{N}_{i}^{m,r}} \exp\left( \tilde{a}_{ij'}^{m,r,h} \right)}\#\left( 52 \right) \end{aligned}$$

The normalized coefficients are used in the attention-weighted neighborhood aggregation defined in main text Eq. 3. The tight-view and loose-view representations are subsequently integrated within each attention head:

$$\begin{aligned} \mathbf{h}_{i}^{m,G,h}=\alpha\mathbf{h}_{i}^{m,tight,h}+\left( 1-\alpha\right)\mathbf{h}_{i}^{m,loose,h}\#\left( 53 \right) \end{aligned}$$

where $\alpha\in\left[ 0,1 \right]$ controls the relative contribution of the two graph views. The representations obtained from $H$ heads are concatenated to form the graph-derived representation:

$$\begin{aligned} \mathbf{h}_{i}^{m,G}={\|}_{h=1}^{H}\mathbf{h}_{i}^{m,G,h}\#\left( 54 \right) \end{aligned}$$

Neighborhood propagation can attenuate intrinsic molecular alterations of individual genes. DRIVE therefore introduces a parallel non-graph branch that directly transforms the original omics profile:

$$\begin{aligned} \mathbf{h}_{i}^{m,NG}=\mathbf{W}_{4}^{m}\phi\left( \mathbf{W}_{3}^{m}\mathbf{f}_{i}^{m}+\mathbf{b}_{3}^{m} \right)+\mathbf{b}_{4}^{m}\#\left( 55 \right) \end{aligned}$$

The non-graph representation $\mathbf{h}_{i}^{m,NG}\in\mathbb{R}^{d}$ is projected into the same latent space as $\mathbf{h}_{i}^{m,G}$. The two branches are integrated to obtain the omics-level gene representation:

$$\begin{aligned} \mathbf{h}_{i}^{m}=\beta\mathbf{h}_{i}^{m,G}+\left( 1-\beta\right)\mathbf{h}_{i}^{m,NG}\#\left( 56 \right) \end{aligned}$$

where $\beta\in\left[ 0,1 \right]$ balances propagated network information and intrinsic molecular evidence. The resulting $\mathbf{h}_{i}^{m}\in\mathbb{R}^{d}$ is supplied to the contrastive multi-omics fusion module.

#### S17.4 The representation learning of omics-common and omics-specific components

The four omics-level representations can contribute differently to individual genes. DRIVE therefore applies a shared attention network to estimate a gene-dependent importance score for each omics branch:

$$\begin{aligned} \tilde{h}_{i}^{m}=\phi\left( \mathbf{W}_{5}\mathbf{h}_{i}^{m}+\mathbf{b}_{5} \right)\mathbf{a}_{1}^{\top}\#\left( 57 \right) \end{aligned}$$

Here, $\mathbf{W}_{5}\in\mathbb{R}^{d_{a}\times d}$, $\mathbf{b}_{5}\in\mathbb{R}^{d_{a}}$ and $\mathbf{a}_{1}\in\mathbb{R}^{d_{a}}$ are shared across all omics branches. The scores are normalised across the omics set $\mathcal{M}$:

$$\begin{aligned} \gamma_{i}^{m}=\frac{\exp\left( \tilde{h}_{i}^{m} \right)}{\sum_{m\mathcal{'\in M}} \exp\left( \tilde{h}_{i}^{m'} \right)}\#\left( 58 \right) \end{aligned}$$

The coefficients satisfy $\gamma_{i}^{m}\in\left[ 0,1 \right]$ and $\sum_{m\mathcal{\in M}} \gamma_{i}^{m}=1$. The omics-common representation is subsequently obtained by the weighted integration in main text Eq. 4. DRIVE then applies the InfoNCE objective in main text Eq. 5, where $\left( \mathbf{h}_{i}^{m},\mathbf{z}_{i,c} \right)$ is the positive pair for gene $v_{i}$, and $\left( \mathbf{h}_{i}^{m},\mathbf{z}_{j,c} \right)$ with $j\neq i$ forms a contrastive negative pair. Negative pairs are defined by gene identity and do not use non-driver annotations, allowing labeled and unlabeled genes to contribute to representation learning.

Omics-specific residual representations and the soft orthogonality regularization are defined in main text Eqs. 6 and 7, respectively. These operations preserve source-specific evidence after removal of the common component and reduce redundancy among the four residual representation spaces.

#### S17.5 Adaptive integration of omics-common and omics-specific representations

After obtaining $\mathbf{z}_{i,c}$ and $\{\mathbf{z}_{i,s}^{m}{\}}_{m\mathcal{\in M}}$, DRIVE forms the candidate representation set $\mathcal{R}_{i}=\{\mathbf{z}_{i,c},\mathbf{z}_{i,s}^{m}\mid m\mathcal{\in M\}}$. A shared scoring function estimates the contribution of the common component:

$$\begin{aligned} s_{i,c}=\phi\left( \mathbf{W}_{6}\mathbf{z}_{i,c}+\mathbf{b}_{6} \right)\mathbf{a}_{2}^{\top}\#\left( 59 \right) \end{aligned}$$

and each omics-specific component:

$$\begin{aligned} s_{i,s}^{m}=\phi\left( \mathbf{W}_{6}\mathbf{z}_{i,s}^{m}+\mathbf{b}_{6} \right)\mathbf{a}_{2}^{\top}, m\mathcal{\in M}\#\left( 60 \right) \end{aligned}$$

Here, $\mathbf{W}_{6}\in\mathbb{R}^{d_{a}\times d}$, $\mathbf{b}_{6}\in\mathbb{R}^{d_{a}}$ and $\mathbf{a}_{2}\in\mathbb{R}^{d_{a}}$ are learnable shared parameters. The common and specific scores are jointly normalised:

$$\begin{aligned} \left[ \lambda_{i,c},\{\lambda_{i,s}^{m}{\}}_{m\mathcal{\in M}} \right]=softmax\left( \left[ s_{i,c},\{s_{i,s}^{m}{\}}_{m\mathcal{\in M}} \right] \right)\#\left( 61 \right) \end{aligned}$$

The coefficients satisfy $\lambda_{i,c}+\sum_{m\mathcal{\in M}} \lambda_{i,s}^{m}=1$. The final fused gene representation is then calculated using main text Eq. 8. This gene-adaptive integration allows the relative contributions of shared and source-specific molecular evidence to vary across genes.

#### S17.6 Driver probability prediction

The final fused representation $\mathbf{z}_{i}\in\mathbb{R}^{d}$ is mapped to a scalar driver probability through a two-layer prediction head:

$$\begin{aligned} \hat{y}_{i}=\sigma\left( \mathbf{w}_{8}^{\top}\phi\left( \mathbf{W}_{7}\mathbf{z}_{i}+\mathbf{b}_{7} \right)+b_{8} \right)\#\left( 62 \right) \end{aligned}$$

Here, $\mathbf{W}_{7}\in\mathbb{R}^{d_{p}\times d}$, $\mathbf{b}_{7}\in\mathbb{R}^{d_{p}}$, $\mathbf{w}_{8}\in\mathbb{R}^{d_{p}}$ and $b_{8}\mathbb{\in R}$ are learnable parameters, and $\sigma\left( \cdot\right)$ is the sigmoid function. The output $\hat{y}_{i}\in\left[ 0,1 \right]$ is used as the DRIVE driver score for gene prioritisation.

#### S17.7 Supervised, self-supervised and joint optimization objectives

The labeled gene set is strongly imbalanced because known drivers constitute a minority of annotated nodes. DRIVE applies focal loss to increase the optimization contribution of difficult and minority-class samples. For each labeled gene $v_{i}\in V_{L}$ with ground-truth label $y_{i}\in\{0,1\}$, the probability assigned to the correct class is:

$$\begin{aligned} p_{i}=y_{i}\hat{y}_{i}+\left( 1-y_{i} \right)\left( 1-\hat{y}_{i} \right)\#\left( 63 \right) \end{aligned}$$

and the class-dependent weight is

$$\begin{aligned} \delta_{i}=y_{i}\delta+\left( 1-y_{i} \right)\left( 1-\delta\right)\#\left( 64 \right) \end{aligned}$$

where $\delta\in\left[ 0,1 \right]$ controls class weighting. The focal loss for gene $v_{i}$ is

$$\begin{aligned} \mathcal{L}_{i}^{focal}=\delta_{i}\left( 1-p_{i} \right)^{\mu}\left( -logp_{i} \right)\#\left( 65 \right) \end{aligned}$$

where $\mu>0$ is the focusing parameter. Candidate driver discovery also requires reliable ranking of genes by predicted probability. DRIVE therefore uses a pairwise margin objective for labeled driver and non-driver gene pairs, denoted by $\left( v_{i},v_{j} \right)$:

$$\begin{aligned} \mathcal{L}_{i,j}^{margin}=max\left( 0,\xi-\hat{y}_{i}+\hat{y}_{j} \right)\#\left( 66 \right) \end{aligned}$$

where $\xi>0$ is the ranking margin. The loss encourages the predicted probability of a known driver to exceed that of a labeled non-driver by at least $\xi$. For pair construction, positive genes are restricted to labeled driver genes in the current training fold, and negative genes are restricted to labeled non-driver genes in the same fold. In each training iteration, 50% of the available labeled non-driver genes are randomly sampled and paired with the labeled driver genes. The pairwise margin loss requires the predicted score $\hat{y}_{i}$ of each driver gene to exceed the predicted score $\hat{y}_{j}$ of the paired non-driver gene by at least the margin threshold ($\xi$=0.1). The averaged focal and pairwise margin components are

$$\begin{aligned} \mathcal{L}^{focal}=\frac{1}{\left| V_{L} \right|}\sum_{v_{i}\in V_{L}} \mathcal{L}_{i}^{focal}\#\left( 67 \right) \end{aligned}$$

$$\begin{aligned} \mathcal{L}^{margin}=\frac{1}{\left| P_{L} \right|\left| N_{L} \right|}\sum_{v_{i}\in P_{L}} \sum_{v_{j}\in N_{L}} \mathcal{L}_{i,j}^{margin}\#\left( 68 \right) \end{aligned}$$

The complete supervised objective is

$$\begin{aligned} \mathcal{L}^{SL}=\mathcal{L}^{focal}+w_{1}\mathcal{L}^{margin}\#\left( 69 \right) \end{aligned}$$

where $w_{1}$ controls the contribution of the pairwise ranking objective.

The contrastive loss $\mathcal{L}_{i}^{cl}$ and orthogonality loss $\mathcal{L}_{i}^{orth}$ provide self-supervised representation signals for all genes, including unlabeled nodes. Their graph-level objective is

$$\begin{aligned} \mathcal{L}^{SSL}=\frac{1}{\left| V \right|}\sum_{v_{i}\in V} \left( \mathcal{L}_{i}^{cl}+w_{2}\mathcal{L}_{i}^{orth} \right)\#\left( 70 \right) \end{aligned}$$

where $w_{2}$ controls the strength of the orthogonality regularisation. Joint optimization follows main text Eq. 9.

### Reference

1.	Schulte-Sasse, R., Budach, S., Hnisz, D. & Marsico, A. Integration of multiomics data with graph convolutional networks to identify new cancer genes and their associated molecular mechanisms. *Nature Machine Intelligence* **3**, 513-526 (2021).

2.	Kipf, T.N. & Welling, M. Semi-supervised classification with graph convolutional networks. *arXiv preprint arXiv:1609.02907* (2016).

3.	Cui, Y.*, et al.* SMG: self-supervised masked graph learning for cancer gene identification. *Briefings in Bioinformatics* **24**, bbad406 (2023).

4.	Li, X., Xu, J., Li, J., Gu, J. & Shang, X. Towards simplified graph neural networks for identifying cancer driver genes in heterophilic networks. *Briefings in Bioinformatics* **26**, bbae691 (2025).

5.	Wu, Y.*, et al.* Deep graph convolutional network-based multi-omics integration for cancer driver gene identification. *Briefings in Bioinformatics* **26**, bbaf364 (2025).

6.	Li, P., Xiao, G., Wang, H., Xu, C. & Zhang, Y. MODCAN: driver gene identification based on multi-omics features and differential co-association networks for tumor subtypes. *BMC Bioinformatics* **27**, 34 (2026).

7.	Cho, S.-P. & Cho, Y.-R. GRAFT: a graph-aware fusion transformer for cancer driver gene prediction. *Briefings in Bioinformatics* **27**(2026).

8.	Wang, H., Lin, C.-X., Zhu, S.-H. & Li, H.-D. MFC-GCN: Identifying Disease Driver Genes via Graph Neural Networks and Multi-Network Fusion Contrastive Learning. in *2025 IEEE International Conference on Bioinformatics and Biomedicine (BIBM)* 1259-1264 (IEEE, 2025).

9.	Su, X.*, et al.* Interpretable identification of cancer genes across biological networks via transformer-powered graph representation learning. *Nature biomedical engineering* **9**, 371-389 (2025).

10.	Li, P. & Xie, M. Multiplex networks-based directed graph neural network for cancer driver gene identification. *PLOS Computational Biology* **22**, e1014275 (2026).

11.	Ying, Z., Bourgeois, D., You, J., Zitnik, M. & Leskovec, J. Gnnexplainer: Generating explanations for graph neural networks. *Advances in neural information processing systems* **32**(2019).
